# Regulation of B Lymphocyte Development by Histone H2A Deubiquitinase BAP1

**DOI:** 10.3389/fimmu.2021.626418

**Published:** 2021-04-12

**Authors:** Yun Hsiao Lin, Yue Liang, HanChen Wang, Lin Tze Tung, Michael Förster, Poorani Ganesh Subramani, Javier M. Di Noia, Simon Clare, David Langlais, Anastasia Nijnik

**Affiliations:** ^1^ Department of Physiology, McGill University, Montreal, QC, Canada; ^2^ McGill Research Centre on Complex Traits, McGill University, Montreal, QC, Canada; ^3^ Department of Human Genetics, McGill University, Montreal, QC, Canada; ^4^ McGill University Genome Centre, Montreal, QC, Canada; ^5^ Institut de Recherches Cliniques de Montréal, Montreal, QC, Canada; ^6^ Department of Medicine, McGill University, Montreal, QC, Canada; ^7^ Department of Medicine, Université de Montréal, Montreal, QC, Canada; ^8^ Department of Biochemistry & Molecular Medicine, Université de Montréal, Montreal, QC, Canada; ^9^ Wellcome Trust Sanger Institute, Hinxton, United Kingdom; ^10^ Department of Microbiology and Immunology, McGill University, Montreal, QC, Canada

**Keywords:** B cells, B cell development, transcriptional regulation, epigenetic regulation, mouse models

## Abstract

BAP1 is a deubiquitinase (DUB) of the Ubiquitin C-terminal Hydrolase (UCH) family that regulates gene expression and other cellular processes, *via* deubiquitination of histone H2AK119ub and other substrates. BAP1 is an important tumor suppressor in human, expressed and functional across many cell-types and tissues, including those of the immune system. B lymphocytes are the mediators of humoral immune response, however the role of BAP1 in B cell development and physiology remains poorly understood. Here we characterize a mouse line with a selective deletion of BAP1 within the B cell lineage (*Bap1* ^fl/fl^ mb1-*Cre*) and establish a cell intrinsic role of BAP1 in the regulation of B cell development. We demonstrate a depletion of large pre-B cells, transitional B cells, and mature B cells in *Bap1* ^fl/fl^ mb1-*Cre* mice. We characterize broad transcriptional changes in BAP1-deficient pre-B cells, map BAP1 binding across the genome, and analyze the effects of BAP1-loss on histone H2AK119ub levels and distribution. Overall, our work establishes a cell intrinsic role of BAP1 in B lymphocyte development, and suggests its contribution to the regulation of the transcriptional programs of cell cycle progression, *via* the deubiquitination of histone H2AK119ub.

## Introduction

B cells are mediators of the humoral immune response, with crucial roles in the defense against infections, anti-tumor immunity, allergic response, and autoimmune disorders (1–3). B cell development takes place in the bone marrow and proceeds through multiple well-characterized developmental stages, guided by the progressive activation of B cell specific transcriptional programs and sequential rearrangement of the immunoglobulin loci. The transcriptional programs of B cell differentiation are controlled by a network of transcription factors E2A, EBF1, PAX5, IKAROS, BCL11A, FOXO1, IRF4, IRF8 and others that work in concert with many histone modifying enzymes and chromatin remodeling complexes (4–7).

Monoubiquitination of histone H2A (H2AK119ub) is a highly abundant histone modification, associated with gene silencing (8, 9). It is primarily deposited on chromatin by the RING1B subunit of the Polycomb repressive complex 1 (PRC1), which acts as a major epigenetic regulator of cell identity, differentiation, and development (9–11). The role of PRC1 in B cell development and lymphomagenesis is well established (10, 12, 13), and the loss of PRC1 complex components in conditional knockout mouse models impairs B cell differentiation and disrupts B cell specific transcriptional programs (14–19). Several deubiquitinases (DUBs) were shown to reverse the monoubiquitination of histone H2A, including BAP1, USP16, MYSM1, USP44, and others (8, 20). However, the role of these DUBs in the transcriptional regulation of B cell development is less well understood. Recent studies demonstrated that MYSM1 and USP16 are essential for B cell development (17, 21–23), while USP44 and USP21 are dispensable (24, 25). At the same time, the function of USP16 as a DUB for histone H2A was questioned, given its primarily cytosolic localization (26). BAP1 is becoming widely recognized as the major ubiquitously expressed DUB for histone H2A, however its role in B cell development and physiology remains poorly explored.

BAP1 is a member of the Ubiquitin C-terminal Hydrolase (UCH) family of DUB proteins. BAP1 forms a complex with Polycomb group proteins ASXL1 and ASXL2, and deubiquitinates histone H2AK119ub to antagonize PRC1-mediated gene silencing (27–30). BAP1 also interacts with host cell factor 1 (HCF1) (31–33), an important transcriptional regulator of cell proliferation and cell cycle progression (34, 35). BAP1 deubiquitinates and stabilizes HCF1, promoting its transcriptional activity (31–33, 35), and in complex with HCF1 also regulates the stability and activity of other transcriptional and metabolic regulators: YY1 (33), KLF5 (36), PGC1α (37), and OGT (37, 38). BAP1 also interacts with forkhead transcription factors FOXK1 and FOXK2, and promotes the transcriptional repression of FOXK2-regulated genes through mechanisms involving H2AK119ub deubiquitination (39, 40). Beyond its major multifaceted roles as a transcriptional regulator, BAP1 is also engaged in the homologous recombination pathway of DNA repair (41), and can localize to the endoplasmic reticulum to deubiquitinate and stabilize inositol-1,4,5-trisphosphate-receptor channel type-3 (IP3R3), modulating Ca^2+^ release and cell apoptosis (42).

BAP1 is an important tumor suppressor in human, with somatic or germinal *BAP1* mutations being common in mesothelioma, uveal melanoma, renal cell carcinoma, and other cancers (43, 44), while mutations in BAP1 interacting Polycomb proteins ASXL1/2 are prevalent in myeloid leukemia (44, 45). In mouse, full systemic loss of BAP1 results in embryonic lethality at mid-gestation (38), while *Bap1* ^+/-^ mice develop normally but have increased incidence of sporadic tumors (46). Importantly, an inducible systemic deletion of *Bap1* in *Bap1* ^fl/fl^ *Cre^ERT2^* mice resulted in broad spectrum hematologic pathology, with an expansion of myeloid leukocytes, and a depletion of platelet and red blood cells, resembling myelodysplastic syndrome (MDS) and chronic myelomonocytic leukemia (CMML) (38). These mice also exhibited impaired thymocyte differentiation and diminished peripheral T cell expansion, with disruptions in the transcriptional programs of cell cycle progression in T cells (47). Importantly, depletion of B cells was also observed following the systemic inducible deletion of *Bap1* in *Bap1* ^fl/f^ *^l^Cre^ERT2^* mice (47). However, this experimental model did not allow characterization of the cell-intrinsic functions of BAP1 in the B cell lineage, independently of BAP1 functions in the precursor hematopoietic stem and progenitor cells (38), and independently of other cells that interact with B cells to regulate their maturation and engagement in immune response (47). Furthermore, BAP1 molecular functions and mechanisms of action in the B cell lineage were not explored in the previous studies. The role of BAP1 in B cell development is the focus of our current work.

Here we characterize the *Bap1* ^fl/fl^mb1-*Cre* mouse strain with a specific loss of BAP1 within the B cell lineage. *Bap1* ^fl/fl^mb1-*Cre* mice showed a systemic reduction in B cell numbers, with a depletion of large pre-B cells, transitional and mature B cell subsets, establishing the cell intrinsic role of BAP1 in the regulation of B cell development. This was associated with a defect in cell cycle progression in *Bap1* ^fl/fl^mb1-*Cre* primary pre-B cells and in *Bap1*-knockout B cell precursor cell lines. RNA-Seq analyses of *Bap1* ^fl/fl^mb1-*Cre* pre-B cells demonstrated a downregulation of genes involved in cell proliferation and cell cycle progression, consistent with both the depletion of the proliferative large pre-B cell subset and impaired cell cycle progression in BAP1 deficiency. ChIP-Seq analyses in the B cell precursor cell line Ba/F3 mapped BAP1 binding across the genome and determined the effects of BAP1 loss on histone H2AK119ub levels, suggesting a direct role of BAP1 in the regulation of genes required for normal cell cycle progression, *via* its deubiquitinase catalytic activity on histone H2A. Overall, our study establishes the non-redundant and cell intrinsic role of BAP1 in the regulation of B cell development.

## Materials and Methods

### Mouse Strains

*Bap1* ^tm1c(EUCOMM)Hmgu^ mouse strain carries a floxed (conditional) allele of *Bap1*-gene and will be referred to here as *Bap1* ^fl^. The strain is derived from ES-cell clone HEPD0526_2_G01; allele structure is provided at https://www.mousephenotype.org/data/alleles/MGI:1206586/tm1c(EUCOMM)Hmgu, and the mice are available from EMMA at: https://www.infrafrontier.eu/search?keyword=Bap1. Specifically, *loxP* sites flank exons 6-12 (ENSMUSE00000121807- ENSMUSE00000121801) of the *Bap1* transcript ENSMUST00000022458.10. We thank the Wellcome Trust Sanger Institute Mouse Genetics Project, its funders, and INFRAFRONTIER/EMMA (www.infrafrontier.eu) for providing this strain. Funding information is found at www.sanger.ac.uk/mouseportal and associated primary phenotypic information at www.mousephenotype.org (48–51). The strain was bred to a transgenic line expressing Cre recombinase under the control of a B cell lineage specific promoter mb1-*Cre* (from Prof. Michael Reth, MPI für Immunbiologie und Epigenetik, Germany) (52). The *Bap1* ^Δ^ allele is predicted to disrupt *Bap1* protein coding sequence from amino acid 126 onward, precluding the expression of full N-terminal UCH catalytic domain and all downstream domains of BAP1 protein. All lines were on the C57BL/6 genetic background. The mice were maintained under specific pathogen-free conditions. Test and control groups were bred as littermates and co-housed together. Experiments were in accordance with the guidelines of the Canadian Council on Animal Care and protocol AUP-2011-6029 approved by the McGill Animal Care Committee. Mice used in experiments were both male and female, 8-16 weeks of age, and always age-matched and sex-matched between the experimental groups. Genotyping was performed with DreamTaq DNA Polymerase (Thermo Fisher Scientific) and primers from IDT Technologies.

### Bone Marrow Transplantation

For competitive bone marrow transplantations, recipient B6.SJL-PtprcaPepcb/Boy mice (JAX002014, congenic for CD45.1) were irradiated with 2 doses of 4.5Gy, delivered 3 hours apart, in a RS2000 irradiator (Rad Source). Wild-type CD45.1-marked bone marrow cells were mixed in a 1:1 ratio with bone marrow cells from mice of *Bap1^fl/+^, Bap1^fl/+^Cre*, or *Bap1^fl/fl^Cre* genotypes, and the mixes transplanted into three independent cohorts of recipient mice. The mice were kept on neomycin in drinking water (2g/l, BioShop) for 3 week, and analyzed at 10 and 17 weeks post-reconstitution.

### Flow Cytometry

Cell suspensions of mouse tissues were prepared in RPMI-1640 (Wisent) with 2% (v/v) FCS, 100μg/ml streptomycin and 100U/ml penicillin (Wisent). The cells were stained for surface-markers in PBS with 2% FCS for 20 minutes on ice, with the following antibodies: APC-conjugated anti-CD43 (S7, BD Biosciences) or anti-IgD (11-26c.2a, BioLegend); APC-eFluor 780-conjugated anti-CD45R/B220 (RA3-6B2, eBioscience) or anti-CD45.1 (A20, eBioscience); Brilliant Violet 650-conjugated anti-CD45R/B220 (RA3-6B2, BioLegend); BUV395-conjugated anti-CD43 (S7, BD Biosciences); eFluor 450-conjugated anti-CD24 (M1/69, eBioscience) or anti-CD45R/B220 (RA3-6B2, eBioscience); FITC-conjugated anti-CD23 (B3B4, eBioscience), anti-CD127/IL7Rα (A7R34, eBioscience), or anti-CD249/BP-1 (6C3, eBioscience); Pacific Blue-conjugated anti-IgD (11-26c.2a, BioLegend); PE-conjugated anti-IgM (II/41, eBioscience); PE-Cy7-conjugated anti-CD19 (6D5, BioLegend), anti-CD21/CD35 (eBio8D9, eBioscience), anti-CD93 (AA4.1, BioLegend), or anti-CD45.2 (104, BioLegend); PerCP-Cy5.5-conjugated anti-CD19 (1D3, Tonbo Biosciences), anti-CD93 (AA4.1, BioLegend), or anti-CD45R/B220 (RA3-6B2, BioLegend); biotin conjugated anti-Pre-B Cell Receptor (SL156, BD Biosciences).

Intracellular staining was performed as previously described (53, 54). The cells were fixed in 2% paraformaldehyde (PFA) in PBS with 2% FCS at 37°C for 10 minutes, and permeabilized in 90% methanol for 30 minutes on ice. The cells were then stained with Alexa Fluor 647-conjugated anti- p53 (1C12, Cell Signaling Technology) and Alexa Fluor 488-conjugated anti-H2A.X Phospho (Ser139) (2F3, BioLegend) antibodies, or FITC-conjugated anti-Ki-67 (B56, BD Biosciences) and Alexa Fluor 647-conjugated Histone H3 Phospho (Ser10) (11D8, BioLegend) antibodies, or the appropriate isotype controls. Viability Dye eFluor^®^ 506 (eBioscience) was used to discriminate dead cells. Annexin V PeCy7 (eBioscience) was used for detection of apoptotic cells. Compensation was performed with BD™ CompBeads (BD Biosciences). The data were acquired on FACS Canto II or BD Fortessa and analyzed with FACS Diva (BD Biosciences) or FlowJo (Tree Star) software.

### B Cell Isolation and Cell Sorting

Total B cells were isolated from mouse spleen *via* magnetic enrichment using EasySep Mouse CD19 Positive Selection Kit II (Stem Cell Technologies), according to the manufacturer’s protocols. B cell subsets were sorted from mouse bone marrow, as previously described (53). Bone marrow was flushed in PBS supplemented with 0.1% BSA and 2mM EDTA, filtered through 40μm cell-strainers, and subjected to red blood cell lysis in ACK buffer (0.15M NH_4_Cl, 10mM KHCO_3_, 0.1mM EDTA). The samples were stained with: biotin-conjugated antibodies against lineage markers [anti-CD3ϵ (145-2C11, BioLegend), anti-CD11b (M1/70, BioLegend), anti-CD11c (N418, BioLegend), anti-Ly-6G/Ly-6C (Gr-1) (RB6-8C5, BioLegend), anti-NK-1.1 (PK136, BioLegend), and anti-TER-119 (TER-119, BioLegend)], followed by APC-eFluor 780-conjugated Streptavidin (eBioscience), Alexa Fluor 488-conjugated anti-IgD (11-26c.2a, BioLegend), APC-conjugated anti-CD43 (S7, BD Biosciences), PE-conjugated anti-IgM (II/41, eBioscience), PE-Cy7-conjugated anti-CD19 (6D5, BioLegend), and PerCP-Cy5.5-conjugated anti-CD45R/B220 (RA3-6B2, BioLegend) antibodies. DAPI was added immediately before sorting for dead cell exclusion. Cell sorting was performed on FACSAria and analyzed with FACS Diva software (BD Biosciences).

### Pre-B Colony Forming Unit (CFU) Assays

Pre-B Colony Forming Unit (CFU) assays with primary mouse bone marrow were performed according to manufacturer’s protocols using MethoCult M3630 media (StemCell Technologies), with 1x10^5^ mouse bone marrow cells plated per dish.

### Western Blotting

Western blotting was performed as previously described (53). Magnetically isolated murine B cells were lysed in RIPA buffer supplemented with protease and phosphatase inhibitors (Thermo Scientific). Protein concentration was measured using the BCA assay (Thermo Scientific). Protein lysate samples were boiled in Laemmli buffer and 1.25% β-mercaptoethanol (Sigma-Aldrich) before loading onto gels, alongside Precision Plus Protein Kaleidoscope standards (Bio-Rad). Upon gel-to-membrane transfer, nitrocellulose membranes (GE Healthcare) were blocked with 5% milk in TBS-T and probed with rabbit monoclonal primary antibodies against BAP1 (D7W7O, Cell Signaling Technology) and β-Actin (D6A8, Cell Signaling Technology) at 4°C overnight, followed by horseradish peroxidase (HRP)-conjugated goat anti-rabbit polyclonal IgG secondary antibody (Abcam) at room temperature for 1 hour, with TBS-T washes after each antibody-incubation. Protein bands were detected using Western Lightning Plus-ECL (PerkinElmer) and HyBlot CL autoradiography films (Harvard Apparatus Canada), according to manufacturer’s protocol.

### RNA Isolation and qRT-PCR

RNA isolation from magnetically isolated B cells was carried out with the EZ-10 DNAaway RNA mini-prep kit (BioBasic) according to the manufacturer’s protocol. The RNA was quantified with Nanodrop spectrophotometry (ThermoFisher Scientific), and reverse transcribed with the M-MLV reverse-transcription kit (Life Technologies, ThermoFisher Scientific). All qPCRs were performed on a StepOnePlus instrument with Power SYBR Mastermix (Applied Biosystems, Life Technologies). The primer sequences for validating *Bap1*-depletion in murine B cells were: *Bap1* -Fw GGA TTG AAA GTC TAC CCA ATT GAT, *Bap1* -Rv CGA GCT TTA TCT GTC CAC TCC T, *Actb* -Fw CTA AGG CCA ACC GTG AAA AG, *Actb* -Rv ACC AGA GGC ATA CAG GGA CA; all primers were purchased from IDT Technologies.

### Cell Culture

Murine B cell lineage precursor cell line Ba/F3 was maintained at 0.5-2 x10^6^ cells/mL in RPMI-1640 (Wisent) with 10% Fetal Calf Serum (FCS, Wisent), 2mM L-Glutamine, 100μg/mL streptomycin, 100U/mL penicillin (Wisent), and 5% WEHI conditioned media as the source of IL-3. Ba/F3 cell lines stably expressing triple-FLAG-tagged murine BAP1 were derived as previously described (53), and maintained under 2μg/mL puromycin selection (Wisent).

### CRISPR-Cas9 Gene Targeting

Plasmid pSpCas9(BB)-2A-GFP (PX458) was obtained from Addgene (#48138). gRNAs targeting *Bap1* were designed using the Zhang lab software (http://crispr.mit.edu): gRNA_*Bap1*_Exon4_ Chr14:32,066,155: gcaaatggatcgaagagcgc and gRNA_*Bap1*_Exon5_Chr14:32,066,654: ggcgtgagtggcacaagagt. The gRNAs were ligated into the BbsI (NEB) digested PX458 plasmid, and the resulting plasmids were transformed into DH5α competent cells, purified using PureLink HiPure Plasmid Filter Midiprep Kit (Invitrogen), and verified by Sanger sequencing. To introduce the constructed plasmid into Ba/F3 cells, nucleofection was performed using Amaxa Cell Line Nucleofector Kit V (Lonza) according to the manufacturer’s protocols. Single GFP^+^ cells were sorted 48 hours after the nucleofection and then expanded over two weeks to generate single cell clones. The loss of BAP1 protein expression in the cell line clones was validated *via* Western blotting, with the following antibodies: anti-BAP1 (D7W7O, Cell Signaling Technology), anti-β-Actin (D6A8, Cell Signaling Technology), HRP-conjugated anti-mouse-Ig (eB144, Rockland), and HRP-conjugated anti-rabbit-Ig (eB182, Rockland).

### AlarmarBlue Assay

Wildtype and *Bap1* ^Δ/Δ^ Ba/F3 cells were seeded into a 96-well plate at a density of 1 × 10^5^/mL, and incubated at 37°C overnight. Subsequently, AlamarBlue (Invitrogen, Thermo Fisher Scientific) was added at 1/10 the total volume to each well and incubated at 37°C for 4 h. Fluorescence intensity was recorded using an EnSpire Plate reader (Perkin Elmer), at the excitation wavelength of 560nm and the emission wavelength of 590nm.

### CFSE Cell Proliferation Assay

Wildtype and *Bap1* ^Δ/Δ^ Ba/F3 cells were washed with PBS, and 5 × 10^6^ cells were resuspended in 1 ml PBS containing 1 μl CellTrace™ CFSE/DMSO solution (Invitrogen, Thermo Fisher Scientific). After 10 min of incubation at 37°C, CFSE was deactivated by adding 12 mL pre-warmed PBS with 5% FBS and incubation at 37°C for 5 min, followed by centrifugation and a second wash with PBS. Subsequently, cells were resuspended in complete culture media at the concentration of 2 × 10^5^/mL, seeded in a 96-well plate at 4 x10^4^ cells per well, and incubated at 37°C overnight. The cells were counterstained with Fixable Viability Dye eFluor™ 780 (eBioscience, Thermo Fisher Scientific). Fluorescence intensity data was acquired on FACS Canto II and analyzed with FACS Diva (BD Biosciences) or FlowJo (Tree Star) software.

### RNA Sequencing (RNA-Seq)

The protocols were as previously described (53). Briefly, RNA was isolated using the MagMAX total RNA kit (Ambion, Life Technologies), and quality assessed using Bioanalyzer RNA Pico chips (Agilent). rRNA depletion and library preparation were performed using the SMARTer Stranded RNA-Seq kit (Takara Clontech). The libraries were sequenced on an Illumina HiSeq 4000 sequencer in paired-end 50bp configuration. The high quality of sequence reads was confirmed using the FastQC tool (Babraham Bioinformatics), and low-quality bases were trimmed from read extremities using Trimmomatic v.0.33 (55). Reads were then mapped to the mouse UCSC mm9 reference assembly using TopHat v2.0.9 in conjunction with Bowtie 1.0.0 algorithm (56–58). Gene expression was quantified by counting the number of uniquely mapped reads with featureCounts using default parameters (59). We retained genes that had an expression level of minimum 5 counts per million reads (CPM) in at least 3 of the samples, and performed quantile normalization with the preprocessCore package to remove batch effects (60). TMM normalization and differential gene expression analyses were conducted using the edgeR Bioconductor package (61). Dimension reduction analysis was performed using the Partial Least Square regression method (62). Pairwise comparisons were performed between samples across different mouse genotypes. Genes with changes in expression ≥ |1.5| fold and Benjamini-Hochberg adjusted *p* values ≤ 0.01 across the three genotypes in either pre-B or immature B cells were considered significant. For data visualization in Integrative Genomics Viewer (IGV) (63), replicates with the same genotype were combined, and bigwig files were generated using a succession of genomeCoverageBed and wigToBigWig tools and scaled per 10 million exon-mapped reads. Gene ontology (GO) and disease ontology enrichment analyses on differentially expressed gene clusters were performed with DAVID Bioinformatics Resources 6.8 (64), and Gene Set Enrichment Analysis (GSEA) was performed in command-line using MSigDB v6.0 with default configuration and permutation within gene sets (65).

### Chromatin Immunoprecipitation (ChIP)

ChIP was performed as described previously (66), with minor modifications. Briefly, cells were fixed with 1% formaldehyde in the culture media for 10 minutes at room temperature, followed by addition of 0.125M of glycine to stop fixation. Nuclei were extracted with 5 minutes lysis in 0.25% Triton buffer, followed by 30 minutes in 200mM NaCl buffer. Nuclei were resuspended in sonication buffer and sonicated for twelve cycles of 30 seconds with a digital sonifier (Branson Ultrasonics) at 80%, with 30 seconds rest in cooled circulating water.

Beads immunocomplexes were prepared overnight by conjugating 40μL of Dynabeads Protein G (Invitrogen, Life Technologies) with antibodies: anti-BAP1 (Cell Signaling Technology, D1W9B, 52.8 μg), anti-FLAG M2 (Sigma, F1804, 5 μg) or anti-H2AK119Ub (Cell Signaling Technology, D27C4, 5 μg). Immunoprecipitation was performed by overnight incubation of antibody-bead matrices with sheared chromatin from the equivalent of 5x10^6^ cells. Four washes were performed with medium-stringency buffers for BAP1-FLAG ChIP, while six washes were performed with low-stringency buffers for histone ChIP and ChIP using the anti-BAP1 antibody (Cell Signaling Technology). Samples were de-crosslinked by overnight incubation at 65°C in 1% SDS buffer, and following RNaseA and Proteinase K enzymatic treatments, ChIP DNA was purified using Qiaquick PCR Cleanup kit (Qiagen).

ChIP-seq libraries were prepared using the Illumina TruSeq kit and sequenced on an Illumina HiSeq 4000 sequencer in paired-end 50bp configuration, with input DNA from the same cells sequenced as negative control. The reads were mapped to the UCSC mouse mm9 reference genome with Bowtie 1.0.0 (57), and BAP1 binding sites identified using peak detection algorithm MACS1.4 (67), with comparisons for read enrichment against control input DNA from the same cells. Normalized sequence read density profiles (bigwig) were generated with Homer tool (68) and visualized with IGV (63). Gene ontology (GO) enrichment analyses on genes associated with BAP1 ChIP-Seq binding clusters were performed on GREAT 4.0.4 (69) with Basal plus extension option, searching for genes within 2kb upstream, 2kb downstream, and 200kb in distal.

### ChIP-Seq and RNA-Seq Data Consolidation

Full gene annotations with transcription start site (TSS) were obtained from the UCSC mouse mm9 reference genome. An in-house Python script was developed to load the genomic locations of ChIP-Seq binding sites and the TSS locations of RNA-Seq dysregulated genes, and search for gene TSS located within a specific distance to each ChIP-Seq binding site. RNA-Seq and ChIP-Seq datasets acquired and described in our study have been deposited into the NCBI public database and are available under GEO Submission GSE162085.

### Statistical Analyses

Statistical analyses used Prism 7.01 (GraphPad Inc.), with Student’s *t*-test for two datasets and ANOVA with Sidak’s or Tukey’s *post hoc* tests for multiple comparisons.

## Results

### Cell Intrinsic Role of BAP1 in B Lymphocyte Development

**Figure 1:**
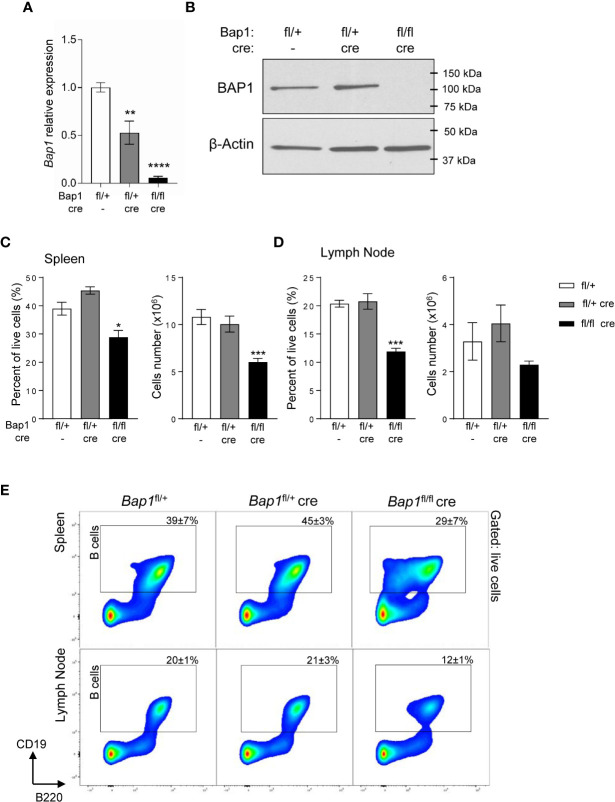
Loss of BAP1 and depletion of B cells in the *Bap1* ^fl/fl^ *Cre* mouse model. **(A)** Reduced *Bap1* transcript levels in B cells magnetically isolated from the total splenocytes of *Bap1* ^fl/fl^ *Cre*, relative to control mice, analyzed by RT-qPCR. Transcript levels are normalized to β-actin, and expressed relative to the transcript levels in control B cells. Data is from 4 mice per group; bars represent mean ± SEM; statistical analysis by ANOVA, **p < 0.01, ****p < 0.0001. **(B)** Loss of BAP1 protein in B cells magnetically isolated from the total splenocytes of *Bap1* ^fl/fl^ *Cre* relative to control mice, analyzed by Western blotting, using β-actin as the loading control; data from one mouse per genotype. **(C–D)** Depletion of B cells in the spleen **(C)** and mesenteric lymph nodes **(D)** of *Bap1* ^fl/fl^ *Cre* relative to control mice, expressed as the percentage of CD19^+^ cells within the total live cells from the tissue and as the absolute cell number. Data presented is from two experiments with 4 mice analyzed per group per experiment. Bars represent mean ± SEM; statistical analysis by ANOVA, NS non-significant, *p < 0.05, ***p < 0.001. **(E)** Representative flow cytometry density plots of the spleen (top) and mesenteric lymph nodes (bottom) stained for B cell lineage markers B220 and CD19, and gated on live cells. CD19^+^ B cell gate is shown and the percentage of cells within the gate for each mouse genotype is presented as mean ± S.D.

To study the role of BAP1 in the B cell lineage, a B cell specific conditional knockout mouse model was generated by crossing the *Bap1*-floxed mouse line *Bap1* ^fl/fl^ to the mb1-*Cre* line expressing *Cre*-recombinase under the control of the B cell lineage specific *Cd79a* gene promoter, from the pre-pro-B cell stage in development (52). As expected, *Bap1* ^fl/fl^ *Cre* offspring were born in normal Mendelian ratios, showed no gross morphological abnormalities, and bred normally. Loss of BAP1 transcript and protein expression was validated in B cells isolated from the spleen of *Bap1* ^fl/fl^ *Cre* and control mice, using qRT-PCR and Western blotting, respectively (**Figures 1A, B**).

To assess the effects of BAP1-loss on the B cell lineage, spleens and mesenteric lymph nodes of *Bap1* ^fl/fl^ *Cre*, *Bap1* ^fl/+^ *Cre*, and control *Bap1* ^fl/+^ mice were analyzed by flow cytometry, gating on CD19^+^ B cells. A significant reduction in the frequency of B cells was observed in the *Bap1* ^fl/fl^ *Cre* relative to control mice in both tissues (**Figures 1C–E**). The absolute numbers of B cells were also significantly reduced in the spleen of *Bap1* ^fl/fl^ *Cre* mice and showed a trend toward reduction in the lymph nodes (**Figures 1C–E**).

We further confirmed the depletion of CD19^+^B220^hi^ B2 lymphocytes in the spleen of *Bap1* ^fl/fl^ *Cre* mice, while CD19^+^B220^lo^ cells were strongly expanded (**Supplemental Figures S1A–C**). Further analysis of the CD19^+^B220^lo^ B cell population identified the expected B1b (CD43^+^CD5^-^) and B1a (CD43^+^CD5^+^) subsets in control mice, while in *Bap1* ^fl/fl^ *Cre* mice the majority of the cells lacked B1 markers expression (CD43^-^CD5^-^, **Supplemental Figures S1D, E**) (70, 71). Overall, we observed a mild depletion of B1b cells (CD19^+^B220^lo^CD43^+^CD5^-^) and expansion of B1a cells (CD19^+^B220^lo^CD43^+^CD5^+^) in *Bap1* ^fl/fl^ *Cre* mice (**Supplemental Figures S1D, E**), while the identity of the CD19^+^B220^lo^CD43^-^ B cells that are strongly expanded in *Bap1* ^fl/fl^ *Cre* mice remains to be further investigated.

Splenic B cells were further analyzed to quantify transitional, follicular (FOL), and marginal zone (MZ) cell populations (**Supplemental Figure S2**) (72). This demonstrated a significant reduction in the numbers of transitional T2-3, follicular FOLI-II, and marginal zone progenitor (MZP) B cells in *Bap1* ^fl/fl^ *Cre* relative to control mice, while the numbers of transitional T1 and MZ B cells were maintained (**Supplemental Figure S2**).

To further validate the cell intrinsic role of BAP1 in B cells, a chimeric mouse model was established. Wild-type CD45.1^+^ marked bone marrow cells were mixed in a 1:1 ratio with bone marrow cells from CD45.2^+^ mice of *Bap1^fl/+^, Bap1^fl/+^Cre*, or *Bap1^fl/fl^Cre* genotypes, and the three mixes transplanted into three independent cohorts of lethally irradiated recipient mice to reconstitute their immune system (**Supplemental Figure S3A**). The recipient mice were analyzed for the relative contribution of CD45.1^+^ and CD45.2^+^ donors to the B cell lineages, by testing the blood at 10 weeks, and the bone marrow and spleen at 17 weeks post-reconstitution. Reduced contribution of the BAP1-deficient *Bap1^fl/fl^Cre* donor cells to the B cell lineage was demonstrated in all the tissues (**Supplemental Figures S3B–E**, **Supplemental Figure S4A**), further confirming the cell intrinsic role of BAP1 in B cells.

### Loss of BAP1 Impairs B Cell Development at the Pre-B Cell Stage in the Bone Marrow

**Figure 2:**
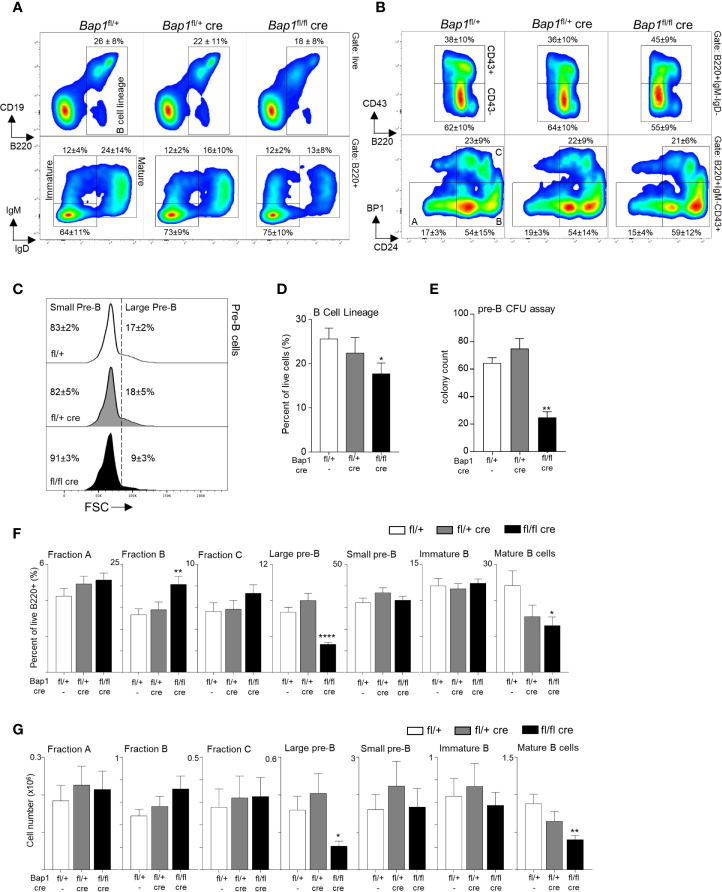
BAP1 loss impairs B cell development from the pre-B cell stage in the bone marrow. **(A, B)** Representative flow cytometry plots of the bone marrow of mice of *Bap1^fl/+^, Bap1^fl/+^Cre*, and *Bap1^fl/fl^Cre* genotypes, demonstrating: (**A**, top) gating on B cell lineage cells (B220^+^); (**A**, bottom) gating on mature B cells (IgM^+^IgD^+^) and immature B cells (IgM^+^IgD^-^); (**B**, top) gating on pre-B cells (B220^+^IgM^-^IgD^-^CD43^-^); (**B**, bottom) gating on Hardy Fractions A, B and C cells as B220^+^IgM^-^IgD^-^CD43^+^, followed by CD24 and BP1 markers. Numbers indicate mean ± S.D. of cell frequency in each gate; data is from 10-12 mice per genotype, consolidated across 3 independent experiments. **(C)** Representative flow cytometry histograms gated on B220^+^IgM^-^IgD^-^CD43^-^ pre-B cells, and showing the gating on large and small pre-B cell subsets based on the forward scatter (FSC) of the cells. Numbers indicate mean ± S.D. of cell frequency in each gate; data is from 10-12 mice per genotype, consolidated across 3 independent experiments. **(D)** Reduced frequency of B lineage cells (B220^+^) in the bone marrow of *Bap1^fl/fl^Cre* relative to control mice. Data is from 10-12 mice per genotype, consolidated across 3 independent experiments. **(E)** Reduction in pre-B cell colony forming units in the bone marrow of *Bap1^fl/fl^Cre* relative to control mice, demonstrating impaired B cell lineage development; colony count is presented per 1x10^5^ mouse bone marrow cells plated per dish; 3 mice were analyzed in total in 2 independent experiments. **(F, G)** Frequencies and absolute numbers of B cell precursor subpopulations in the bone marrow of *Bap1^fl/fl^Cre* and control mice; the cells were gated as live B220^+^ cells followed by IgM^-^IgD^-^CD43^+^CD24^lo^BP1^lo^ for Fraction A, IgM^-^IgD^-^CD43^+^CD24^+^BP1^lo^ for Fraction B, IgM^-^IgD^-^CD43^+^CD24^+^BP1^+^ for Fraction C, IgM^-^IgD^-^CD43^-^FSC^hi^ for large pre-B cells, IgM^-^IgD^-^CD43^-^FSC^lo^ for small pre-B cells, IgM^+^IgD^-^ for immature B cells, and IgM^+^IgD^+^ for mature B cells. Bars represent mean ± SEM; flow cytometry data is from 10-12 mice per genotype, consolidated across 3 independent experiments, with cell counts presented per one tibia and femur. Statistical analyses were performed with ANOVA; *p < 0.05, **p < 0.01, ****p < 0.0001.

To analyze the impact of BAP1 loss on B cell development and to pinpoint the specific developmental transitions dependent on BAP1, the bone marrow of *Bap1^fl/fl^Cre* and control mice was analyzed by flow cytometry. This demonstrated a significant reduction in the frequency of B cells in the *Bap1^fl/fl^Cre* relative to control mice (gated as B220^+^, **Figures 2A–D**). The defect in B cell development in *Bap1^fl/fl^Cre* mice was further confirmed with a colony forming units (CFU) assay, demonstrating a reduction in the numbers of pre-B CFUs in *Bap1^fl/fl^Cre* bone marrow samples (**Figure 2E**). Further characterization of B cell progenitor subpopulations demonstrated a significant depletion in the frequencies and absolute numbers of large pre-B cells and mature B cells, and an expansion of Fraction B pro-B cells in *Bap1^fl/fl^Cre* relative to control bone marrow (**Figures 2F, G**). No changes in the abundance of the common lymphoid progenitors, multipotent progenitors, and hematopoietic stem cells were observed (data not shown), further confirming the specificity of the *Cre* transgene and the phenotype to the B cell lineage. Overall, our findings indicate the cell intrinsic role of BAP1 as a regulator of B cell development, and demonstrate that BAP1 loss impacts pre-B cells, as well as transitional and mature B cell subsets.

### Impaired Cell Viability and Cell Cycle Progression in BAP1-Deficient Pre-B Cells

**Figure 3:**
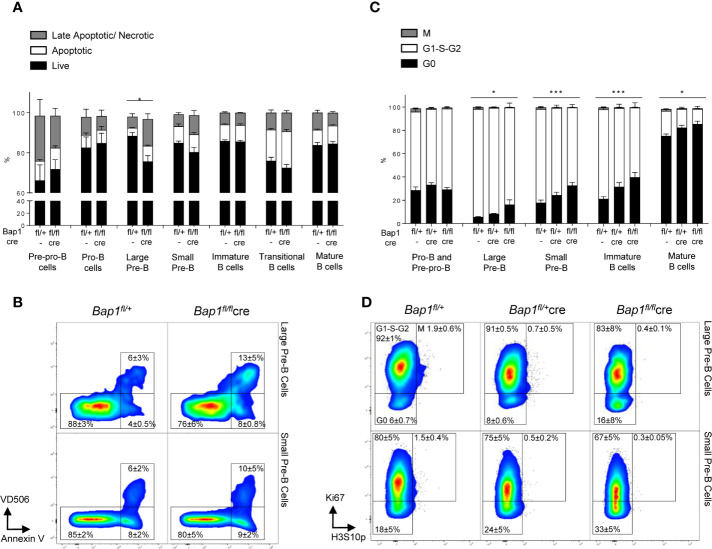
Impact of *Bap1*-deficiency on the B cell lineage cell viability and cell cycle progression. **(A, B)** Impaired viability of large pre-B cells in *Bap1^fl/fl^Cre* mice. Bone marrow and splenocytes of mice of *Bap1^fl/+^* and *Bap1^fl/fl^Cre* genotypes were stained with Annexin V to identify apoptotic cells and with Viability Dye V506 (eBioscience) to identify necrotic cells, as well as for cell surface markers to identify mature splenic B cells (B220^+^IgM^+^IgD^+^), transitional splenic B cells (B220^+^IgM^+^IgD^-^), immature bone marrow B cells (B220^+^IgM^+^IgD^-^), small pre-B cells (B220^+^IgM^-^IgD^-^CD19^+^CD43^-^FSC^lo^), large pre-B cells (B220^+^IgM^-^IgD^-^CD19^+^CD43^-^FSC^hi^), pro-B cells (B220^+^IgM^-^IgD^-^CD19^+^CD43^+^), and pre-pro-B cells (B220^+^IgM^-^IgD^-^CD19^-^CD43^+^). **(A)** Frequency of live (AnxV^-^V506^-^), early apoptotic (AnxV^+^V506^-^), and late apoptotic/necrotic (AnxV^+^V506^+^) cells within each B cell subpopulation in *Bap1^fl/fl^Cre* and control mice. Bars represent mean ± SEM; n=4 mice were analyzed per genotype in total over two independent experiments; statistical analysis used ANOVA, *p < 0.05. **(B)** Representative flow cytometry density plots, gated on the large and small pre-B cells and showing the reduced frequency of live cells and increased frequency of late apoptotic/necrotic cells for the large pre-B cells in *Bap1^fl/fl^Cre* mice. Cell frequency in each gate is indicated as mean ± S.D. **(C, D)** Impaired cell-cycle progression in *Bap1^fl/fl^Cre* B cells. Bone marrow of mice of *Bap1^fl/+^, Bap1^fl/+^Cre*, and *Bap1^fl/fl^Cre* genotypes was stained for Ki67 and histone H3S10p, to identify G0 (Ki67^-^H3S10p^-^), G1-S-G2 (Ki67^+^H3S10p^-^), and M phase (Ki67^+^H3S10p^+^) cells. **(C)** Frequency of G0, G1-S-G2, and M phase cells within each B cell subpopulation, showing impaired cell cycle progression from the pre-B stage in development onward in *Bap1^fl/fl^Cre* mice. Bars represent mean ± SEM; n=4 mice were analyzed per genotype in total over two independent experiments; statistical analyses were performed using ANOVA; *p < 0.05, ***p < 0.001, or not significant if not indicated. **(D)** Representative flow cytometry density plots, gated on large and small pre-B cells and showing elevated frequency of G0 cells and reduced frequency of G1-S-G2 cells in *Bap1^fl/fl^Cre* mice; cell frequency in each gate is indicated as mean ± S.D.

To assess how the loss of BAP1 affects B cell development and physiology, we analyzed the effects of BAP1 loss on cell viability and cell cycle progression throughout the B cell lineage, with flow cytometry. This demonstrated a specific reduction in the viability of large pre-B cells, caused by an increase in the proportion of late apoptotic/necrotic cells within this B cell subset (**Figures 3A–B**).

To further analyze the effects of BAP1 loss on cell cycle progression, bone marrow samples from *Bap1^fl/fl^Cre* and control mice were stained for Ki67 and histone H3S10p, to identify cells in G0 (Ki67^-^H3S10p^-^), G1-S-G2 (Ki67^+^H3S10p^-^), and M (Ki67^+^H3S10p^+^) phases of the cell cycle within each B cell subpopulation. This revealed a significant increase in the fraction of G0 cells and a corresponding decrease in G1-S-G2 cells within the large pre-B cell, small pre-B cell, immature, and mature B cell subpopulations in *Bap1^fl/fl^Cre* mice (**Figures 3C, D**, **Supplemental Figures S4B, C**), indicating impaired cell proliferation and cell cycle progression. In contrast no differences were observed in the cell cycle state of pre-pro-B and pro-B cells (**Figure 3C**). Overall, this indicated that impaired cell survival and proliferation contribute to the depletion of pre-B cells and possibly to the broader defect in B cell lineage development in *Bap1^fl/fl^Cre* mice.

**Figure 4:**
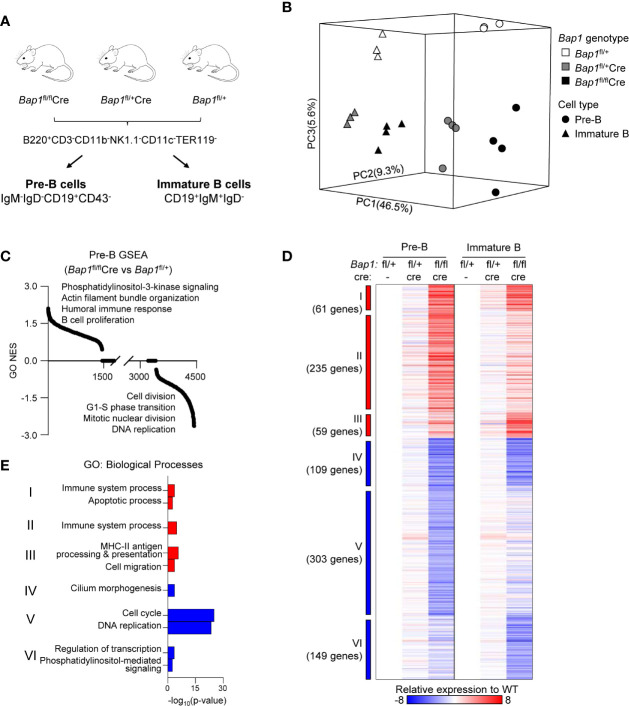
RNA-Seq analysis of the transcriptome of *Bap1*-deficient pre-B and immature B cells. Data presented is from 3-4 mice per genotype. **(A)** Schematic representation of the mouse genotypes and cell sorting protocols for the RNA-Seq experiment. **(B)** 3D principal component analysis model demonstrating the gene expression profiles of each RNA-Seq sample: differences between cell types are described by principal component 1 (PC1, 46.5% variability) and differences between genotypes are described by PC2 and PC3 (9.3% and 5.6% variability, respectively). **(C)** Normalized enrichment scores (NES) of 4,436 pre-established biological processes expression signatures used in the gene set enrichment analysis (GSEA). **(D)** Heat map displaying 916 significantly dysregulated genes when comparing *Bap1* ^fl/fl^ *Cre* and control *Bap1* ^fl/+^ cells. The significance threshold is: fold change ≥ 1.5 and False Discovery Rate (FDR) ≤0.01. Relative expressions to the average of pre-B and immature B control *Bap1* ^fl/+^ group are used to generate the heat map. Genes are grouped into Clusters I-VI based on their expression pattern across the two cell types and three genotypes. **(E)** Gene ontology (GO) enrichment analysis on the genes from the Clusters I-VI described in **(D)**. The top two enriched biological processes terms per cluster are displayed.

We further observed a reduction in the expression of IL7Rα on the surface of large pre-B cells, but not other B cell precursor subsets in *Bap1^fl/fl^Cre* relative to control mice (**Supplemental Figures S5A, B**), while the expression of pre-B cell receptor (pre-BCR) on large and small pre-B cells was unchanged (**Supplemental Figures S5C, D**). Subsequent RNA-seq analyses of *Bap1* ^fl/fl^ *Cre* and control pre-B cells (**Figure 4**), showed no difference in the expression of *Il7r* gene or surrogate light chain gene *Igll1* in *Bap1* ^fl/fl^ *Cre* pre-B cells (**Table S1A, B**, *Vpreb1-2* did not reach >5CPM expression threshold for quantification). This indicates that the differences in IL7Rα protein expression on pre-B cell surface are likely mediated *via* indirect mechanisms, and are not a result of direct regulation of its encoding gene by BAP1.

Given the previously reported roles of BAP1 not only in transcriptional regulation but also in DNA repair (41), we further analyzed the B cell precursor cell populations in the bone marrow of *Bap1^fl/fl^Cre* and control mice for the levels of γH2AX as a marker of DNA damage (73) and p53 protein as a marker of DNA damage response activation (74). There were no differences in the levels of γH2AX or p53 between *Bap1^fl/fl^Cre* and control cells for any of the B cell precursor cell populations (**Supplemental Figure S6**). This suggested that the role of BAP1 as a regulator of B lymphocyte development is likely independent of its functions in DNA repair and is primarily linked to its role as a transcriptional and epigenetic regulator of gene expression.

### RNA-Seq Analysis of BAP1-Deficient B Cell Precursors

To further understand the functional impact of BAP1 loss on B cell development, RNA-Seq gene expression analysis was conducted on live pre-B and immature B cells, isolated from the bone marrow of *Bap1* ^fl/fl^ *Cre*, *Bap1* ^fl/+^ *Cre*, and control *Bap1* ^fl/+^ mice through FACS-sorting (**Figure 4A**, **Table S1A, B**). Loss of *Bap1* ^fl^ exon expression was confirmed in *Bap1* ^fl/fl^ *Cre* datasets (**Supplemental Figure S7A**). Dimension reduction analysis of gene expression profiles showed clear segregation of the two cell types (PC1 - 46.5% variance) and the three genotypes (PC2 - 9.3% variance, PC3 - 5.6% variance), and indicated major transcriptional changes in the BAP1-deficient cells (**Figure 4B**). To explore the biological functions dysregulated within the transcriptome of BAP1-deficient B cells, a gene set enrichment analysis (GSEA) (65) was performed and highlighted the dysregulation of the transcriptional signatures linked to “cell proliferation”, “DNA replication”, and “cell division”, as well as many other biological processes in *Bap1* ^fl/fl^ *Cre* cells (**Figure 4C**, **Table S1C**).

Differential gene expression analysis across the three *Bap1* genotypes was performed, at fold change ≥1.5 and false discovery rate (FDR) ≤0.01. This identified 916 genes significantly dysregulated in *Bap1* ^fl/fl^ *Cre* relative to control *Bap1* ^fl/+^ cells (**Figure 4D**, **Table S1A, B**). In contrast, in *Bap1* ^fl/+^ *Cre* cells the downregulation of *Cd79a* was the only transcriptional change to reach the significance threshold, and is likely due to the direct effect of *Cre* knockin on the *Cd79a* locus (52). This demonstrates the very limited effects of *Cre* or heterozygous *Bap1* loss on the transcriptome, and confirms that the major transcriptional changes in *Bap1* ^fl/fl^ *Cre* are due to the loss of BAP1 function.

The dysregulated genes were segregated into Clusters I-VI, based on their upregulation or downregulation status in *Bap1* ^fl/fl^ *Cre* versus control samples across the two cell types (**Figure 4D**). Gene ontology (GO) enrichment analysis was performed for the genes in each cluster, to explore the biological functions dysregulated in the BAP1*-*deficient B cell precursors. The upregulated transcriptional signatures, represented by Clusters I-III, showed a mild enrichment for GO-terms “immune system process”, “cell migration” and “apoptotic process” (**Figure 4E**, **Supplemental Table S1D**). As an example the upregulated genes included several receptors for chemokines and other messengers (*Cxcr7*, *Cxcr5*, *Cr2*, *IL2rg*, *Il9r*, *S1pr3*, *CD40*, *Ahr*), some pattern recognition receptors (*Tlr1*, *Tlr9*, *Naip5*, *Clec12a*), transcriptional regulators involved in immune cell development and activation (*Irf5*, *Irf7*, *Irf8*, *Nfkb2*), both pro-survival and pro-apoptotic Bcl-family proteins (*Bcl2*, *Bbc3*), and some oncogenes (*Myc*). In contrast, the downregulated transcriptional signature of BAP1-deficient pre-B cells (Cluster V) showed a highly significant enrichment of GO-terms “cell cycle progression” and “DNA replication” (**Figure 4E**, **Supplemental Table S1D**), indicating the downregulation of genes involved in cell proliferation and cell cycle progression. This is consistent with the depletion of the highly proliferative large pre-B cell subset within the cell population (**Figures 2F, G**), but also with the previously reported functions of BAP1 in the regulation of transcriptional programs of cell cycle progression in other cell types (32, 34–36, 47). Overall, we hypothesize that a disruption in cell proliferation and cell cycle progression may contribute to the defects in B cell development in BAP1-deficiency.

### Impaired Cell Proliferation in BAP1-Deficient B Cell Precursor Cell Lines

**Figure 5:**
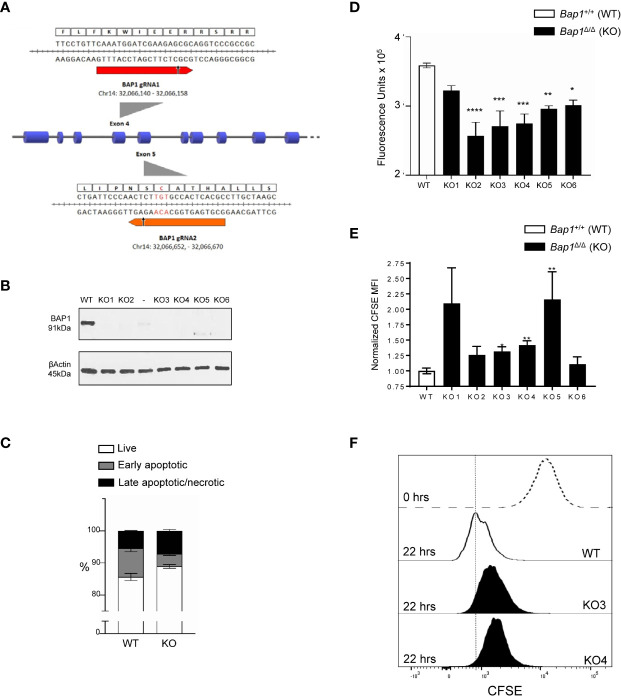
Impaired proliferation in CRISPR/Cas9 BAP1-deficient B cell precursor cell line Ba/F3. **(A)** gRNA targeted cleavage on *Bap1* gene sequence. gRNA1 and gRNA2 were designed to target exons 4 and 5 of mm9 *Bap1* gene, respectively. The gRNA-targeted DNA fragments of exons 4 and 5, as well as the corresponding amino acids of BAP1 protein are displayed in detail. Arrows indicate predicted sites of Cas9 mediated double strand break (DSB). Both the cleavage sites are upstream of the key catalytic residue of BAP1 protein Cys-91 (red). **(B)** Loss of BAP1 protein expression in *Bap1^Δ/Δ^* cell line clones 1-6, demonstrated with Western blotting, using β-Actin as the loading control. Additional clone with residual BAP1 expression, analyzed on the gel between clones 2 and 3, was excluded from subsequent analysis. **(C)** Cell viability analysis with flow cytometry, staining the cells with Annexin V and Viability Dye, to identify live (AnxV^-^V506^-^), early apoptotic (AnxV^+^V506^-^), and late apoptotic/necrotic (AnxV^+^V506^+^) cells; consolidated data from 6 *Bap1^Δ/Δ^* cell clones is presented. **(D)** Cell proliferation analysis with colorimetric alamarBlue assay, demonstrating reduced proliferation of *Bap1^Δ/Δ^* cell clones. Data presented in from one experiment and was reproduced in a second independent experiment. **(E)** Cell proliferation analyses with a flow cytometry based CFSE-dilution assay, gating on live cells. Consolidated data from two independent experiments is presented, normalizing the CFSE mean fluorescence intensity (MFI) of *Bap1^Δ/Δ^* cell clones in each experiment to their corresponding WT control. In **(C–E)**, bars represent mean ± SEM; statistical analyses were performed using ANOVA; *p < 0.05, **p < 0.01, ***p < 0.001, ****p < 0.0001, or not significant if significance is not indicated. **(F)** Representative histograms showing CFSE fluorescence of WT and *Bap1^Δ/Δ^* clones KO3 and KO4, at 22 hours of culture, gating on live cells. A histogram showing CFSE fluorescence of WT cells prior to culture (0 hours, dotted line) is included as a positive control.

To further analyze the impact of BAP1 loss on B cell survival and proliferation and the molecular mechanisms involved, *Bap1* gene was targeted with CRISPR/Cas9 in the B cell precursor cell line Ba/F3. Two gRNAs were designed to target *Bap1* exons 4 and 5 that encode the key N-terminal catalytic domain of the protein (**Figure 5A**). Cell clones were screened by PCR for deletions within the *Bap1* locus, and six clones were selected and further analyzed by Western blotting to confirm the loss of BAP1 protein (**Figure 5B**). There was no defect in the viability of *Bap1* ^Δ/Δ^ cell lines, as demonstrated by equivalent frequencies of live, early apoptotic, and late apoptotic/necrotic cells between the *Bap1* ^Δ/Δ^ and control cell cultures (**Figure 5C**). The effect of BAP1 loss on the proliferation of the B cell lines was further analyzed with two approaches: a colorimetric alamarBlue assay conducted in bulk cell population, and a flow cytometric CFSE-dilution assay providing proliferation data at single cell resolution. Both approaches demonstrated a reduction in cell proliferation for all the *Bap1* ^Δ/Δ^ clones tested, reaching statistical significance for the majority of the clones (**Figures 5D–F**). This further confirmed the role of BAP1 in the regulation of B cell proliferation, and indicated the *Bap1* ^Δ/Δ^ Ba/F3 cell lines as a model for further analyses of the molecular mechanisms involved.

### Mapping BAP1 Binding Across the B Cell Genome

**Figure 6:**
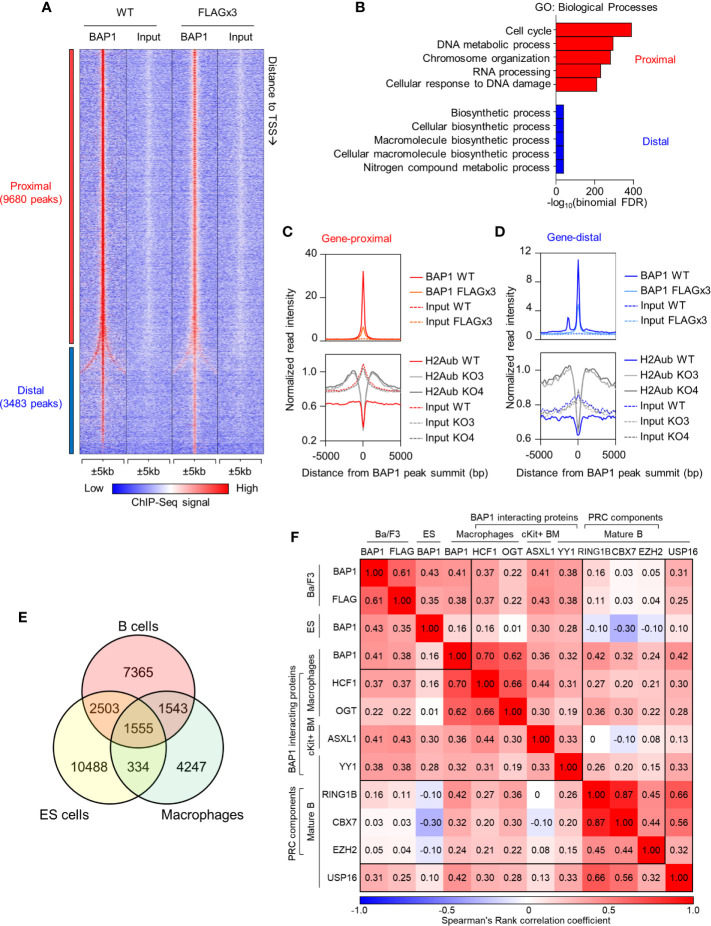
ChIP-Seq analysis of BAP1 binding sites, and the impact of BAP1 loss on histone H2AK119ub levels, in the B cell precursor cell line Ba/F3. **(A)** Heat map showing the tag density of 13,163 BAP1 binding sites identified in ChIP-Seq experiments from wild type Ba/F3 B cell lineage precursor cells (WT) and Ba/F3 cells stably expressing triple-FLAG-tagged BAP1 (FLAG x3). The sites are ranked based on their distance to the nearest gene transcriptional start site (TSS). Gene-proximal sites are defined as having the nearest gene TSS within 1kb, and gene-distal sites are defined as having the nearest gene TSS further than 1kb. **(B)** Gene Ontology analysis of the nearest genes to each BAP1 binding site, performed on the GREAT website (69). The -log_10_(binomial FDR) value for each term is plotted. **(C, D)** Histograms showing the average normalized tag densities of BAP1 and histone H2AK119ub around the gene-proximal **(C)** and gene-distal **(D)** BAP1 binding sites. The tag densities of BAP1 are determined from the ChIP-Seq data of WT and FLAG x3 Ba/F3 cells, and the tag densities of histone H2AK119ub are from the ChIP-Seq data of WT and two *Bap1^Δ/Δ^* (KO) Ba/F3 cells lines. **(E)** Venn diagram comparing the BAP1 binding sites identified in our ChIP-Seq datasets from B cells with BAP1 binding sites identified in previous studies in ES-cells and macrophages (38, 75). **(F)** Analyses of the co-localization of BAP1 binding sites identified in our ChIP-Seq data with the binding sites for other functionally related transcriptional regulators in previously published studies. Heatmap displaying the Spearman rank correlations between all pairwise comparisons for the BAP1 ChIP-Seq data and the ChIP-Seq datasets of other functionally related transcriptional regulators. Spearman correlations were calculated using the normalized tag densities ( ± 100bp around peak summit) across the entire set of binding sites identified from the ChIP-Seq experiments. The BAP1 ChIP-Seq from Ba/F3 cells represents our datasets and is described in **(A, B)**. The others are public datasets, downloaded and re-analyzed using our pipeline. These include the BAP1 ChIP-Seq from ES cells (75), the BAP1, HCF1, and OGT ChIP-Seq from bone marrow derived macrophages (38), the ASXL1 ChIP-Seq from c-Kit+ bone marrow cells (76), the RING1B, CBX7, EZH2, and USP16 ChIP-Seq from quiescent CD43^-^ resting splenic B cells (17), and the YY1 ChIP-Seq from follicular B cells (CD19^+^AA4^-^CD21^lo^CD23^hi^) (77); all datasets are from mouse.

To identify the genes directly regulated by BAP1, we mapped the genome-wide DNA-binding sites of BAP1 by ChIP-Seq. The experiment was carried out in the B cell precursor cell line Ba/F3: a) using wild type cells with an anti-BAP1 antibody, and b) using cells stably expressing 3xFLAG-tagged BAP1 protein with an antibody against the FLAG epitope (38). We identified a total of 13,163 BAP1 binding sites (or peaks) across the genome, with a very high concordance between the two datasets (**Figure 6A**, **Supplemental Table S1E**). Ordering the BAP1-binding sites based on their distance to the nearest gene transcription start site (TSS), 74% were located within 1kb of the nearest TSS and were therefore classified as gene-proximal (≤1kb to TSS, 9,680 sites), and the remaining 26% were classified as gene-distal (>1kb to TSS, 3,483 sites) (**Supplemental Table S1E**). To gain functional insight into the transcriptional targets of BAP1 in B cells, gene ontology analysis was performed on the genes in the vicinity of each BAP1 binding site using the Genomic Regions Enrichment of Annotations Tool (GREAT) (69). This demonstrated that the genes near the gene-proximal BAP1 binding sites were highly enriched for biological process ontology terms “cell cycle” and “DNA metabolic process” (**Figure 6B**, **Supplemental Table S1F**), suggesting the direct involvement of BAP1 in the regulation of the transcriptional programs required for B cell proliferation.

### BAP1 Functions as a Deubiquitinase for Histone H2AK119ub in B Cells

To gain insights into the mechanisms of BAP1 transcriptional and epigenetic regulation in the B cell lineage, we performed ChIP-Seq analysis for the repressive histone mark H2AK119ub, comparing the *Bap1* ^Δ/Δ^ and control Ba/F3 B cell precursor cell lines. Two clones of *Bap1* ^Δ/Δ^ cells were selected, KO3 and KO4, based on full loss of BAP1 protein and a proliferation defect representing an average of that seen across all the available clones (**Figures 5D, E**). The ChIP-Seq data demonstrated that on average the genomic regions surrounding the BAP1 binding sites had low levels of histone H2AK119ub in wild type Ba/F3 cells, consistent with their predominant localization at promoters of expressed genes (**Figures 6C, D**). Importantly, the levels of histone H2AK119ub at these BAP1-bound genomic sites were significantly elevated in *Bap1* ^Δ/Δ^ relative to wild-type cells (**Figures 6C, D**, **Supplemental Figure S7B**). This indicated the non-redundant role of BAP1 as a deubiquitinase for histone H2AK119ub in the B cell lineage, as previously also reported for other cell types (27–30).

To compare the roles of BAP1 in transcriptional regulation in B cells and other cell types, we consolidated our BAP1 ChIP-Seq and previously published BAP1 ChIP-Seq datasets, with macrophages and ES cells being the source of the available data (38, 75). Spearman rank correlation analysis was performed for all pairwise comparisons. The results demonstrate a considerable overlap in the genomic location of BAP1 binding across the cell types, and also highlight a subset of BAP1 binding sites potentially specific to B cells (**Figures 6E, F**, **Supplemental Figures S7C–D**, full lists of sites available in **Supplemental Table S1E**). Genes nearest to each of the “shared” and the “cell-type specific” subsets of BAP1 binding sites were further analyzed for enrichment of biological process GO-terms, demonstrating shared BAP1 binding at the genes involved in many housekeeping biological processes, including “cell-cycle”, across the different cell lineages (**Supplemental Figure S7C**).

To gain mechanistic insights into BAP1 cross-talk with other transcriptional regulators, we further consolidated our BAP1 ChIP-Seq with ChIP-Seq datasets for BAP1-associated transcriptional regulators ASXL1 (27–30), HCF1 (31–34), OGT (37, 38), and YY1 (33), for components of PRC complexes RING1B, CBX7, and EZH2 (9–11), and with a dataset for deubiquitinase USP16 (17), using data from murine B cells (where available) and also datasets from macrophages and hematopoietic progenitors (17, 38, 76, 78). A significant correlation in genomic localization was observed between BAP1 and BAP1-interacting proteins ASXL1, HCF1, YY1 and to a lesser extent OGT (**Figure 6F**, **Supplemental Figure S7D**, **Table S1E**), suggesting that BAP1 acts in cooperation with these proteins in the B cell lineage, as in other cell types (27–34, 37, 38). Weak correlation was observed between the genomic localization of BAP1 and the RING1B catalytic subunit of the histone H2A ubiquitin ligase PRC1, and no correlation was seen with the PRC2 catalytic subunit EZH2 (**Figure 6F**, **Supplemental Figure S7D**, **Table S1E**), supporting the antagonistic roles of BAP1 and PRCs in transcriptional regulation. Interestingly, a positive correlation was observed between BAP1 and USP16, indicating that these deubiquitinases may target some common genomic locations, and suggesting cooperative or complementary functions (**Figure 6F**, **Supplemental Figure S7D**, **Table S1E**).

### Exploring BAP1 Regulated Transcriptional Programs in B Cell Development

**Figure 7:**
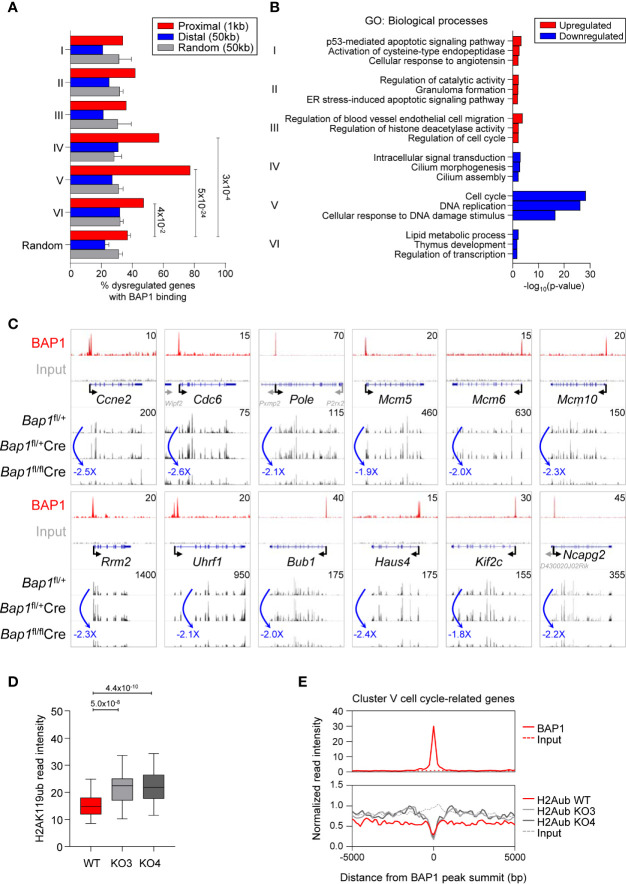
BAP1 in the regulation of genes involved in cell proliferation and cell cycle progression in pre-B cells. **(A)** Consolidation of the RNA-Seq and ChIP-Seq datasets, described in **Figures 4** and **6**, showing the percentages of genes dysregulated in expression in *Bap1^fl/fl^Cre* cells with at least one BAP1 binding site within the indicated range of their TSS. Analysis is performed for each of the Cluster I-VI of the genes dysregulated in *Bap1^fl/fl^Cre* cells, as defined in **Figure 4D**. The random genes cluster consists of ten groups of 300 genes randomly selected from 24,421 genes in the mm9 genome. The random binding sites consist of ten groups of 10,000 genomic locations randomly selected from the mm9 genome. Fisher Exact Test is used to calculate the *p*-values. **(B)** Gene ontology (GO) enrichment analysis for the Clusters I-VI genes, dysregulated in expression in *Bap1^fl/fl^Cre* cells, and containing a proximal or distal BAP1 binding site. Top three enriched biological process terms per Cluster are displayed. **(C)** Genomic snapshots of selected genes that represent the putative direct transcriptional targets of BAP1 in pre-B cells. The genes are selected from Cluster V genes that are downregulated in expression in *Bap1^fl/fl^Cre* pre-B cells, carry a proximal or distal BAP1 binding site, and are functionally linked to cell proliferation and cell cycle progression. ChIP-Seq tracks for BAP1 and input DNA are shown on the top two lanes. The gene feature track is shown in the middle. Averaged RNA-Seq tracks for pre-B cells are in the bottom three lanes, with fold changes comparing expression levels in *Bap1* ^fl/fl^ *Cre* and control *Bap1* ^fl/+^ samples indicated. The maximum data range of each track is indicated at the top-right corner of the track. **(D)** Box plot showing histone H2AK119ub read intensities ±2.5kb to BAP1 binding sites near putative BAP1-regulated Cluster V genes, functionally linked to cell proliferation and cell cycle progression; (GO terms: GO:0007049 ~cell cycle; GO:0006260 ~DNA replication; GO:0051301 ~cell division; mmu04110: Cell cycle; and/or mmu03030: DNA replication, 69 genes in total, **Table S1G**). The Mann-Whitney U test was used to calculate the *p*-values. **(E)** Histograms showing the average normalized read intensities of BAP1 and histone H2AK119ub at the BAP1 binding sites proximal to the select subset of Cluster V genes, functionally linked to cell proliferation and cell cycle progression, as specified above. The read intensities of BAP1 are determined from the ChIP-Seq data of WT and FLAG x3 Ba/F3 cells, and the read intensities of histone H2AK119ub are from the ChIP-Seq data of WT and two *Bap1^Δ/Δ^* (KO) Ba/F3 cell lines.

We consolidated our ChIP-Seq and RNA-Seq datasets and identified in total 591 genes that had BAP1 binding sites in their proximity and were dysregulated in expression in BAP1 deficiency (**Supplemental Table S1G**). Repeating the analysis for each cluster of genes dysregulated in BAP1-deficient B cells, we observed a strong and significant overrepresentation of BAP1 binding sites at the genes in the downregulated Clusters IV-VI (**Figure 7A**, **Figures 4D, E**). In particular, the strongest overrepresentation of genes with BAP1 binding sites was seen for Cluster V, enriched for the genes involved in cell cycle progression and downregulated in expression in BAP1-deficient pre-B cells (**Figure 7A**, **Figures 4D, E**). Overall, this demonstrates a strong concordance between our RNA-Seq and ChIP-Seq datasets, consistent with the primary function of BAP1 as a transcriptional activator. The strong correlation between BAP1 occupancy, histone H2AK119ub levels, and gene expression suggests a direct role of BAP1 in the regulation of genes essential for normal cell cycle progression in pre-B cells.

Gene ontology (GO) analysis further confirmed that the set of genes in proximity of BAP1 binding sites and downregulated in expression in BAP1-deficient pre-B cells was strongly enriched for GO-terms “cell cycle” and “DNA replication” (Cluster V, **Figure 7B**, **Supplemental Table S1H**). Notably, this gene set included the key genes encoding: regulators of G1/S cell cycle transition (*Ccne2*, *Cdc25a*) and the initiation of DNA replication (*Cdc6*, *Cdc45*, *Cdt1*); components of the core DNA replication machinery (*Pola1*, *Pold1*, *Pole*, *Pole2*, *Pcna*, *Rfc1*, *Dna2*, *Lig1*, *Mcm2-7*) and enzymes involved in deoxyribonucleotide synthesis (*Rrm1*, *Rrm2*); regulators of G2/M cell cycle transition (*Bub1*, *Cdc25a*, *Espl1*, *Plk1*), mitotic spindle dynamics (*Haus4-5*, *Ndc80*, *Kif2c*, *Kif23*, *Kif11*, *Kntc1*), and cytokinesis (*Anln*, *Ect2*, *Prc1*); key regulators of chromatin structure through the S and M phases of the cell cycle (*Esco2*, *Ncapd2*, *Ncapg2*, *Uhrf1*); and several general biomarkers of cell proliferation (*Foxm1*, *Mki67*) (**Figure 7C**, **Supplemental Figure S7E**, **Table S1G**). Overall, our data suggests a direct role of BAP1 in the regulation of the transcriptional programs of cell proliferation, linked to its requirement for cell cycle progression in pre-B cells and for normal B cell development.

To gain insights into the putative mechanisms through which BAP1 may regulate the transcriptional programs of cell cycle progression in B cell development, we assessed the levels of the repressive histone mark H2AK119ub in *Bap1* ^Δ/Δ^ relative to control Ba/F3 B cell precursor cell lines from our ChIP-Seq data, specifically focusing on the BAP1 binding sites within the promoters Cluster V genes downregulated in expression in BAP1 deficient pre-B cells and assigned GO-terms “cell cycle”, “DNA replication”, and “cell division” (69 genes, **Supplemental Table S1G**). Importantly, a significant increase in histone H2AK119ub levels was observed in *Bap1* ^Δ/Δ^ relative to control cells at these promoters (**Figures 7D, E**). This strongly suggests that BAP1 regulates the expression of the genes essential for cell proliferation and cell cycle progression in B cell development at least in part *via* the deubiquitination of histone H2AK119ub.

## Discussion

In this work, we establish the non-redundant and cell intrinsic role of BAP1 in the normal progression of B cell development, and suggest BAP1 as a regulator of the transcriptional programs required for cell proliferation and cell cycle progression in pre-B cells, *via* its deubiquitinase catalytic activity for histone H2AK119ub.

B cell dysfunction was previously reported by Arenzana et.al., following an inducible deletion of *Bap1* in the *Bap1* ^fl/fl^ *Cre^ERT2^* mouse model (47), showing a depletion of pre-pro-B, small pre-B, immature, and mature B cell subsets. It is, however, important to note the complementarity and the differences between our experimental systems, with Arenzana et al. analyzing an acute and systemic loss of BAP1 (38, 47), whereas our current work assesses BAP1 deletion specifically within the B cell lineage (52). Such differences likely account for the differential depletion of the B cell precursor subsets across the two models. Overall, our work for the first time allows to assess the cell intrinsic role of BAP1 following B cell lineage commitment, independently of its role in the earlier hematopoietic progenitor cells or the cells of the bone marrow niche (38, 47).

The full mechanisms leading to the depletion of large pre-B cells in *Bap1* ^Δ/Δ^ mice remain to be further explored. The crosstalk between IL7R and pre-BCR signaling is the major regulator of the balance between large pre-B cell proliferation, their further differentiation to small pre-B cells, and the subsequent initiation of Ig light chain rearrangement (79–81). Our study therefore suggests a possible role for BAP1 as a transcriptional and epigenetic regulator of cell cycle progression downstream of IL7R and pre-BCR signaling. Future studies should address whether *Bap1*-deficiency impairs the proliferative response of pro-B and pre-B cells to ex vivo IL7 stimulation (82, 83), and also test for direct protein interactions between BAP1 and the components of IL7R and pre-BCR signaling machinery. pre-BCR signaling and large pre-B cell proliferation are also required to establish a normal B cell repertoire diversity and for the negative selection of autoreactive B cell clones (79). The impact of *Bap1*-deficiency on repertoire diversity and the negative selection of autoreactive B cells therefore remains to be further addressed.

While our study links the depletion of large pre-B cells in *Bap1* ^fl/fl^ mb1-*Cre* mice to the disruption in cell proliferation and the direct role of BAP1 in the regulation of genes required for cell cycle progression, the expansion of Fraction B pro-B cells also seen in *Bap1* ^fl/fl^ mb1-*Cre* mice is not linked to changes in cell cycle or cell survival, and remains to be further explored in future work. Similarly, the mechanisms leading to the depletion of mature B cells in the bone marrow, spleen, and lymph nodes of *Bap1* ^fl/fl^ mb1-*Cre* mice remain to be further addressed and may be distinct from the mechanisms driving the depletion of large pre-B cells, analyzed in our current study. In particular, the cross-talk between BAP1 functions in mature B cells and the BAFF/BAFFR pathway essential for the survival of transitional-2, follicular and marginal zone B cells (84, 85), and the NOTCH pathway required for marginal zone B cell development (86), merit further investigation. Interestingly, the role of BAP1 as a regulator of NOTCH pathway signaling is already indicated in other cell types (87). Furthermore, the role of BAP1 in the development and maintenance of B1 cells is not sufficiently addressed in our current work, and requires further investigation, given that B1 cell development is driven by distinct progenitor cells, occurs in distinct developmental waves during ontogeny, and is regulated by distinct transcription factors and gene expression networks (71, 88).

Our work has advanced the understanding of the molecular functions of BAP1 within the B cell lineage. Our findings suggest that BAP1 may have a direct role in the regulation of the transcriptional programs of cell cycle progression in B cell development. Although our transcriptional analyses of *Bap1* ^fl/fl^ *Cre* pre-B cells is somewhat confounded by the depletion of the more proliferative large pre-B cell subset from the analyzed cell population, it is important to note that the dysregulation in cell cycle was observed in both large pre-B and small pre-B cell subsets that make up the pre-B cell pool sorted for the transcriptional profiling. Furthermore, there was a highly significant overrepresentation of BAP1 binding sites in proximity of the genes dysregulated in our transcriptional data from *Bap1* ^fl/fl^ *Cre* pre-B cells, providing further support for our conclusions. The overrepresentation of BAP1 binding sites was especially significant at the downregulated genes involved in cell proliferation and cycle progression, and the loss of BAP1 resulted in increased H2AK119ub levels at these sites, supporting a direct role of BAP1 and its deubiquitinase catalytic activity for histone H2A in their transcriptional regulation. Overall, we hypothesize that a disruption in BAP1-regulated transcriptional programs of cell proliferation and cell cycle progression may contribute to the defects in B cell development in BAP1-deficiency.

The functional link between the loss of BAP1 and impaired cell proliferation, suggested for the B cell lineage in our current work, is shared across several different cell types (32, 34–36, 47). As already discussed, an inducible systemic deletion of *Bap1* in mice results in impaired proliferation of thymocytes and peripheral CD4 T cells (47), suggesting shared BAP1 function across the different cell types of the lymphoid lineage. Depletion of erythrocytes and platelets was also reported in this model, however not linked to specific cell proliferation defects (38). BAP1 was also shown to be essential for proliferation of several non-hematopoietic cell lines, regulating transcriptional programs of cell cycle progression in complex with HCF1 (31–36). Importantly, however, the role of BAP1 as a positive regulator of cell maintenance and proliferation is not universal, and *Bap1* deletion in mice results in an expansion of myeloid leukocytes and progenitor cells, resembling the pathology of MDS and CMML (38). The apparently opposing roles of BAP1 in the cells of myeloid and lymphoid cell lineages is surprising, especially as our analyses demonstrate a strong correlation in the genomic location of BAP1 binding sites across the genome of B cells, macrophages, and ES-cells (38, 75), suggesting common transcriptional functions. The mechanisms resulting in the distinct outcomes of BAP1 loss on the myeloid and lymphoid cell lineages merit further investigation, and may provide insights into the functions of BAP1 and its ASXL binding partners as tumor suppressors (43–45).

We demonstrate that the loss of BAP1 results in an increase in histone H2AK119ub levels at BAP1 genomic binding sites in B cell precursor cells, both globally across the genome and also locally at the promoters of BAP1-target genes involved in the regulation of B cell proliferation. Overall, this indicates that the role of BAP1 in the B cell lineage is closely coupled to its activity as a deubiquitinase for histone H2AK119ub. Deposition of the repressive histone mark H2AK119ub on chromatin by PRC1 is traditionally linked to long-term silencing of developmentally regulated genes, to control the pathways of cellular differentiation and lineage specification (9–11). More recently the role of PRC1 and H2AK119ub in the regulation of other transcriptional programs has emerged, such as the pro-survival and pro-apoptosis transcriptional programs (89), and the programs of cell proliferation and cell cycle progression (90–92) across different cell types. Our findings further highlight the putative role of the H2AK119ub epigenetic mark and the machinery that regulates its deposition on and removal from chromatin in the regulation of the transcriptional programs of cell proliferation in B lymphocytes.

BAP1 is an important tumor suppressor, and germline *BAP1* mutations in human result in a strong predisposition to a range of cancers, including mesothelioma, uveal melanoma, renal cell carcinoma, and others (43, 44). Although B cell lymphomas are not commonly associated with *BAP1* mutations, cases of non-Hodgkin lymphoma have been reported in carriers of germline *BAP1* mutations (93). Furthermore, lymphomas that carry other genetic aberrations have been shown to acquire *BAP1* gene silencing *via* epigenetic mechanisms (94). This raises the possibility that BAP1 may function as a tumor suppressor also in the B cell lineage. This hypothesis remains to be further tested in mouse models, for example by screening for the incidence of spontaneous lymphomas in *Bap1^fl/fl^ Cre* mice, or by tracking other age-associated phenotypes and the life-span of the animals. Future studies will need to further address these questions, providing insights into potential tumor suppressor functions and other functions of BAP1 within the B cell lineage.

The molecular mechanisms underlying the tumor suppressor activities of BAP1 remain controversial, especially in the light of this and many other reports implicating BAP1 as a positive regulator of cell proliferation (35). The tumor suppressor activity of BAP1 in myeloid progenitor cells was linked to its antagonism with PRC2, as the transformation of BAP1-deficient myeloid cells was associated with an increase in PRC2-mediated transcriptional repression and could be abolished by the loss or inhibition of the PRC2 catalytic subunit EZH2 (95, 96). Similar mechanisms are implicated in the tumor suppressor activities of BAP1 in mesothelioma (95), but not in uveal melanoma cell lines (97). Other mechanisms linking BAP1 molecular functions and its tumor suppressor activity are also proposed, including: its roles in transcriptional regulation of pro-survival and pro-apoptosis transcriptional programs in cross-talk with PRC1 (89); its role as a positive regulator of ferroptosis *via* repression of *SLC7A11* gene expression (98); its role in the transcriptional regulation of cellular metabolic programs (37, 99–101), and in the regulation of Ca^2+^ signaling and apoptosis *via* deubiquitination and stabilization of receptor-channel IP3R3 (42, 102). We observed that *SLC7A11* was not expressed in pre-B and immature B cells, from either control or *Bap1^fl/fl^Cre* mice, indicating that the regulation of ferroptosis by BAP1 is unlikely to play significant physiological role in these B cell precursor cells. Our data further revealed no significant correlation in the genomic localization of BAP1 binding sites and the binding sites of the PRC2 catalytic subunit EZH2 in the B cell lineage. Future studies will need to further address the molecular mechanisms underlying the tumor suppressor activities of BAP1 in different cell types, in the light of its apparent positive role in the regulation of cell proliferations in non-transformed cells of many tissues.

We have demonstrated that the loss of BAP1 results in impaired B lymphocyte development, and suggested the link of this phenotype to the altered transcriptional profile and the defects in proliferation in BAP1-deficient pre-B cells. It is important to note, however, that BAP1 remains expressed throughout the B cell lineage, and that cell cycle analysis in *Bap1^fl/fl^ Cre* mice revealed significant changes not only in pre-B cells, but also in immature and mature B cells. It is therefore highly likely that BAP1 remains engaged as an important transcriptional regulator throughout B cell ontogeny. Cell proliferation is essential not only for the formation and maintenance of the pool of naïve B cells, but also for the normal execution of B cell mediated immune response, including clonal expansion during the germinal center reaction (103–105). Future studies should address the cell intrinsic role of BAP1 in B cells during the execution of humoral immune response, including the germinal center reaction, and the differentiation and persistence of plasma and memory B cells.

In summary, our work establishes the non-redundant and cell intrinsic role of BAP1 in B cell development. It further suggests a direct role of BAP1 in the induction of the transcriptional programs of cell proliferation and cell cycle progression in pre-B cells, linked to its catalytic function as a deubiquitinase for histone H2AK119ub.

## Data Availability Statement

The RNA-Seq and ChIP-Seq datasets have been uploaded to public repository: GEO Submission GSE162085.

## Ethics Statement

The animal study was reviewed and approved by McGill University Animal Care Committee, protocol AUP-2011-6029.

## Author Contributions

Experiments were designed by AN, YHL, YL, DL, and MF., and the experimental work was carried out and data acquired by YHL and YL, with assistance from HW, LTT, MF, and AN. Bioinformatics data analyses were performed by HW and YL, with the supervision of DL. SC provided the mouse line, genotyping protocols, and scientific advice for the studies. JN and PS provided valuable scientific advice and reagents for the project, including the protocols for the derivation of CRISPR/Cas9 cell lines, and valuable input on data analyses and interpretation. The manuscript was written by AN, YHL, YL, and HW.

## Funding

The work was funded by the Natural Sciences and Engineering Research Council of Canada (NSERC) Discovery Grant RGPIN-2016-05657, and supported by the Canadian Foundation for Innovation. AN is a Canada Research Chair Tier II in Hematopoiesis and Lymphocyte Differentiation. YHL is a recipient of a Doctoral Training Award from the Fonds de Recherche du Québec Santé (FRQS) and a Cole Foundation Studentship, and was previously supported by the Frederick Banting Tri-Council Graduate Scholarship. YL is a recipient of Internal Scholarship from McGill Faculty of Medicine. YL and LTT were previously supported by the Richard Birks Fellowship from the Department of Physiology of McGill University. HW was a recipient of an FRQS Masters Training Studentship. MF was a recipient of an International Postdoctoral Fellowship from the German Research Association (DFG FO 900/1-1) and a Cole Foundation Postdoctoral Fellowship. DL is funded by FRQS and the Canadian Institutes for Health Research (CIHR). JN is a Merit Research Fellow and PS is a Doctoral Fellow from FRQS.

## Conflict of Interest

The authors declare that the research was conducted in the absence of any commercial or financial relationships that could be construed as a potential conflict of interest.
