# Supplementary material for: Regulation of B Lymphocyte Development by Histone H2A Deubiquitinase BAP1
Source: Front Immunol. 2021 Apr 12;12:626418. doi: 10.3389/fimmu.2021.626418 (PMC8072452; doi:10.3389/fimmu.2021.626418)
Supplement: Supplementary file 1 [file DataSheet_1.pdf]

Supplemental Figure S1. Analysis of B2 and B1 cell populations in *Bap1*$^{fl/fl}$ *Cre* mice.
(A-B) Absolute numbers of CD19^{+}B220^{hi} and CD19^{+}B220^{lo} B lymphocytes in the spleen and mesenteric lymph nodes of *Bap1*$^{fl/fl}$ *Cre* and control mice. **(C)** Representative flow cytometry plots of the spleen of *Bap1*$^{fl/fl}$ *Cre* and control mice, gated on live cells, and showing CD19^{+}B220^{hi} and CD19^{+}B220^{lo} B lymphocyte gates. Percentages of cells within each gate are presented as mean ± S.D. **(D)** Absolute numbers of CD19^{+}B220^{lo}CD43^{+}CD5^{-} B1b and CD19^{+}B220^{lo}CD43^{+}CD5^{+} B1a lymphocytes in the spleen of *Bap1*$^{fl/fl}$ *Cre* and control mice. **(E)** Representative flow cytometry plots of the spleen of *Bap1*$^{fl/fl}$ *Cre* and control mice, gated on live CD19^{+}B220^{lo} cells, and showing CD43^{+}CD5^{-} B1b and CD43^{+}CD5^{+} B1a lymphocytes (Wells et al., 1994; Baumgarth, 2011). Data is from 3-5 mice per genotype per experiment, with 2 experiments performed in (A-C) and one experiment in (D-E). Percentages of cells within each gate are presented as mean ± S.D. Bars represent mean ± SEM; * $p<0.05$, ** $p<0.01$, *** $p<0.001$, comparisons by *t*-test for two groups and by ANOVA for multiple comparisons. Related to Figure 1.

Figure S1

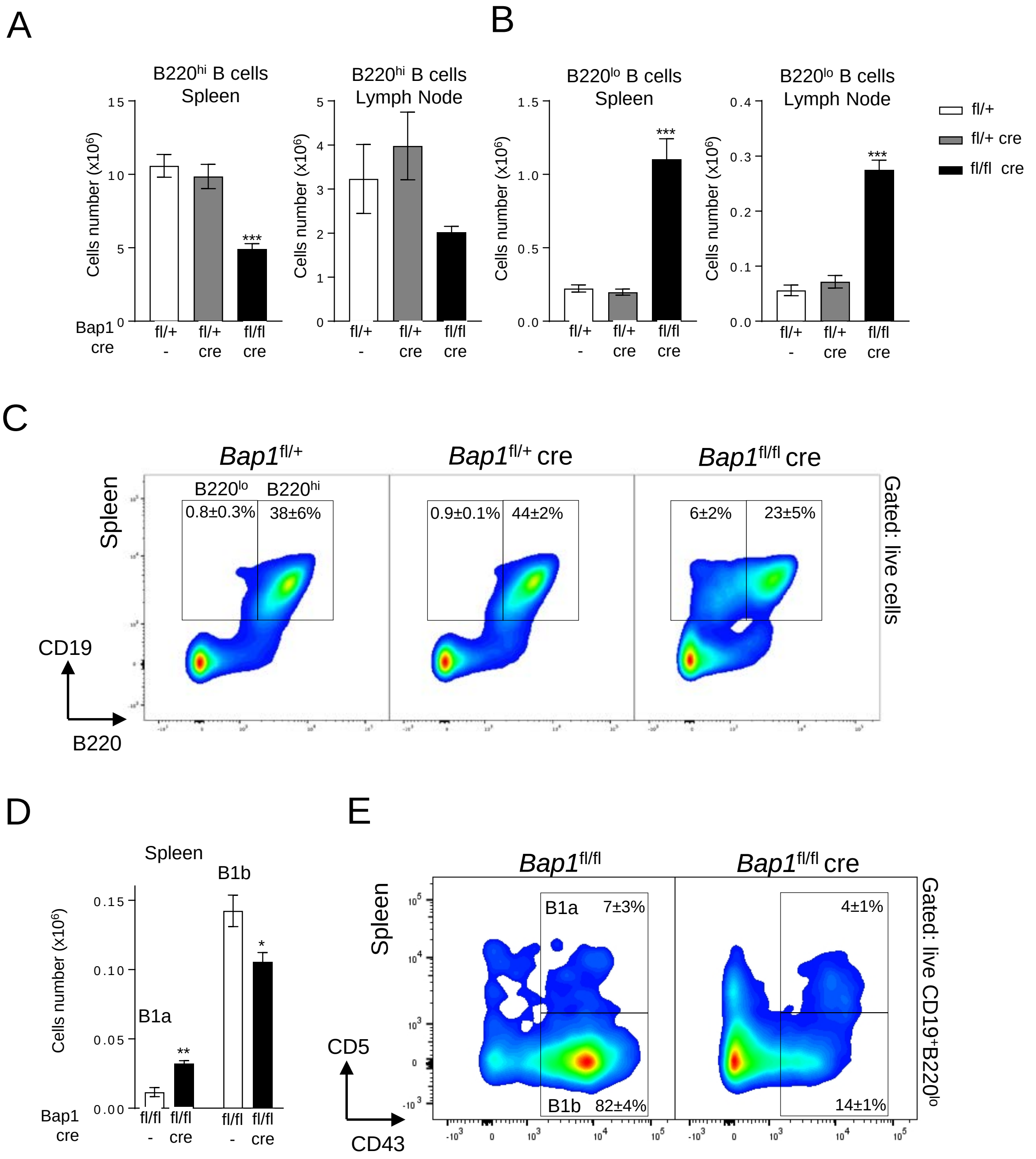
A
B220hi B cells Spleen
B220hi B cells Lymph Node
Cells number (x10^6)
Bap1 fl/+ fl/+ fl/fl
cre - cre cre
***
B
B220lo B cells Spleen
B220lo B cells Lymph Node
fl/+
fl/+ cre
fl/fl cre
C
Bap1fl/+
Bap1fl/+ cre
Bap1fl/fl cre
Spleen
B220lo
B220hi
0.8±0.3%
38±6%
0.9±0.1%
44±2%
6±2%
23±5%
Gated: live cells
CD19
B220
D
Spleen
B1a
B1b
Cells number (x10^6)
Bap1 fl/fl fl/fl fl/fl fl/fl
cre - cre - cre
**
*
E
Bap1fl/fl
Bap1fl/fl cre
Spleen
B1a 7±3%
4±1%
B1b 82±4%
14±1%
CD5
CD43
Gated: live CD19+B220lo

Supplemental Figure S2. Analysis of splenic B cell populations in *Bap1*$^{fl/fl}$ *Cre* mice.
(A-B) Quantification of transitional T1, T2, and T3, follicular FOLI and FOLII, marginal zone progenitor (MZP) and marginal zone (MZ) B cell populations in the spleen of *Bap1*$^{fl/fl}$ *Cre* and control mice, presented **(A)** as a percentage of live CD19^{+} B cell lineage splenocytes, and **(B)** as an absolute cell number per mouse spleen. Cells are gates as live CD19^{+} B cells, followed by B220^{+}CD93^{+}IgM^{+}CD23^{-} for T1, B220^{+}CD93^{+}IgM^{+}CD23^{+} for T2, and B220^{+}CD93^{+}IgM^{lo}CD23^{+} for T3 transitional B cells, B220^{+}CD93^{-}CD21^{+}IgM^{+}IgD^{+} for FOLI and B220^{+}CD93^{-}CD21^{+} IgM^{hi}IgD^{+} for FOLII follicular B cells, B220^{+}CD93^{-}CD21^{hi}IgM^{hi}CD23^{+} for MZP and B220^{+}CD93^{-}CD21^{hi}IgM^{hi}CD23^{-} for MZ B cells (Allman and Pillai, 2008). Data is from 4-5 mice per genotype. Bars represent mean ± SEM; statistical analysis by *t*-test; * p<0.05, ** p<0.01, *** p<0.001. **(C)** Representative flow cytometry plots of the spleen of *Bap1*$^{fl/fl}$ *Cre* and control mice, showing the gating for T1, T2, T3, FOLI, FOLII, MZ, and MZP B cell populations, according to the markers listed above. Percentages of cells within each gate for each mouse genotype are presented as mean ± S.D. Related to Figure 1.

Figure S2

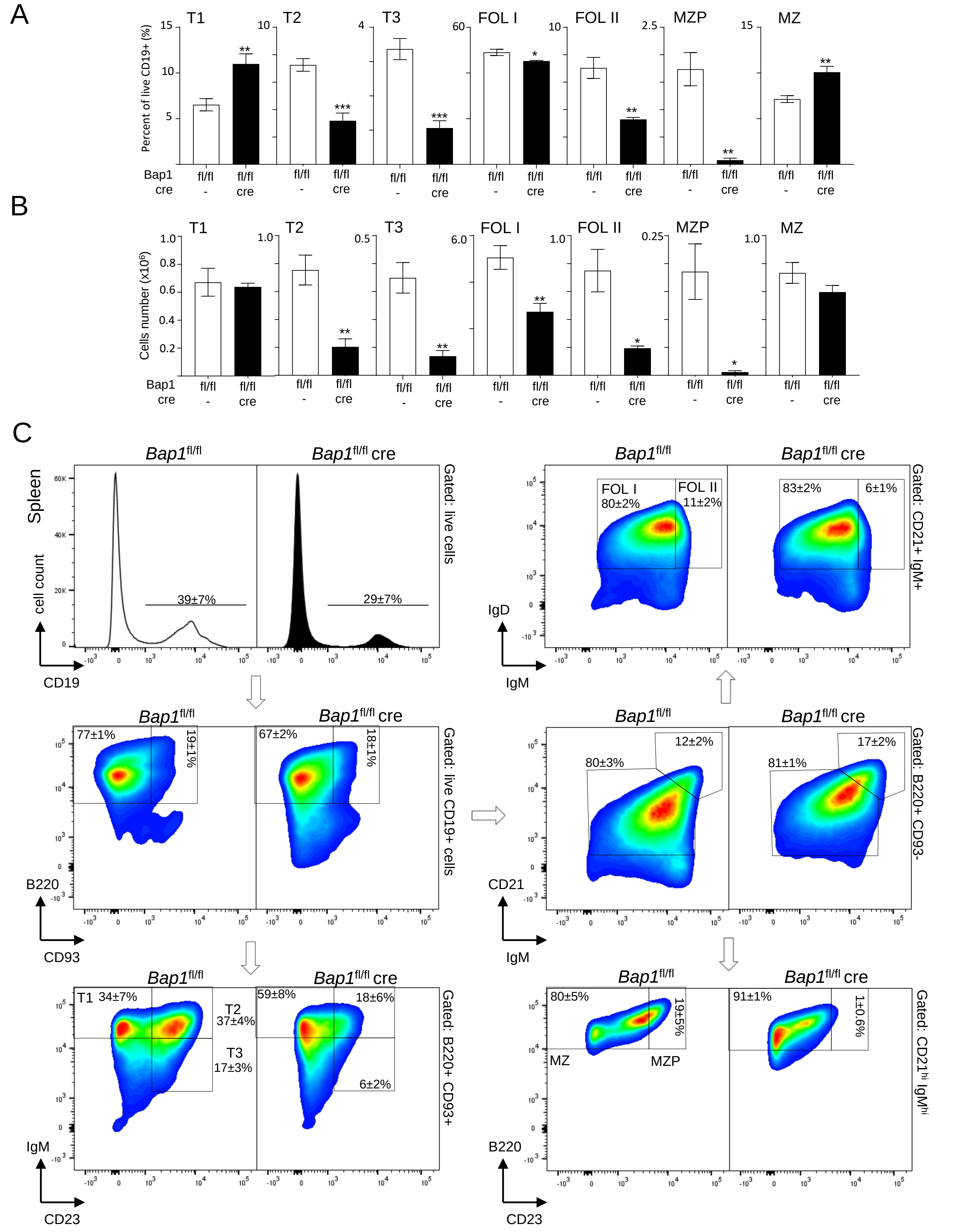
A
T1
T2
T3
FOL I
FOL II
MZP
MZ
Percent of live CD19+ (%)
Bap1
cre
fl/fl
-
fl/fl
cre
B
Cells number (x10^6)
C
Spleen
cell count
CD19
Bap1fl/fl
Bap1fl/fl cre
39±7%
29±7%
Gated: live cells
B220
CD93
77±1%
19±1%
67±2%
18±1%
Gated: live CD19+ cells
IgM
CD23
T1 34±7%
T2 37±4%
T3 17±3%
59±8%
18±6%
6±2%
Gated: B220+ CD93+
IgD
IgM
FOL I 80±2%
FOL II 11±2%
83±2%
6±1%
Gated: CD21+ IgM+
CD21
IgM
80±3%
12±2%
81±1%
17±2%
Gated: B220+ CD93-
B220
CD23
80±5%
19±5%
MZ
MZP
91±1%
1±0.6%
Gated: CD21hi IgMhi

Supplemental Figure S3. Cell intrinsic role of BAP1 in B cells demonstrated in a chimeric mouse model.

(A) Schematic diagram of the competitive bone marrow transplantation experiment. Wild-type CD45.1-marked bone marrow cells were mixed in a 1:1 ratio with bone marrow cells from mice of *Bap1*$^{fl/+}$, *Bap1*$^{fl/+}$*Cre*, or *Bap1*$^{fl/fl}$*Cre* genotypes, and the mixes transplanted into three independent cohorts of lethally irradiated recipient mice to reconstitute their immune system. Reduced contribution of *Bap1*$^{fl/fl}$*Cre* donor cells to the B cell lineage is demonstrated with the analysis of blood, bone marrow, and spleen of the recipient mice, at 10 weeks and 17 weeks post-reconstitution, respectively. **(B)** Representative flow cytometry density plots, of the blood, bone marrow, and spleen of the recipient mice, gating on live B cells, as CD19^{+} cells in the blood, B220^{+} cells in the bone marrow, and B220^{+} or B220^{lo}IgM^{+} cells in the spleen. Gates indicate B cells derived from the CD45.2^{+} test and the CD45.1^{+} competitor bone marrow donors; mean ± S.D. of cell frequency in each gate for all mice within the group is indicated. **(C-E)** Bar charts showing data quantification for **(C)** blood, **(D)** bone marrow, and **(E)** spleen. Bars represent mean ± SEM, data is from n≥4 recipient mice per group, with mice in each recipient group injected with same mix of donor bone marrow cells; statistical comparisons with ANOVA, *** p<0.001.

Figure S3

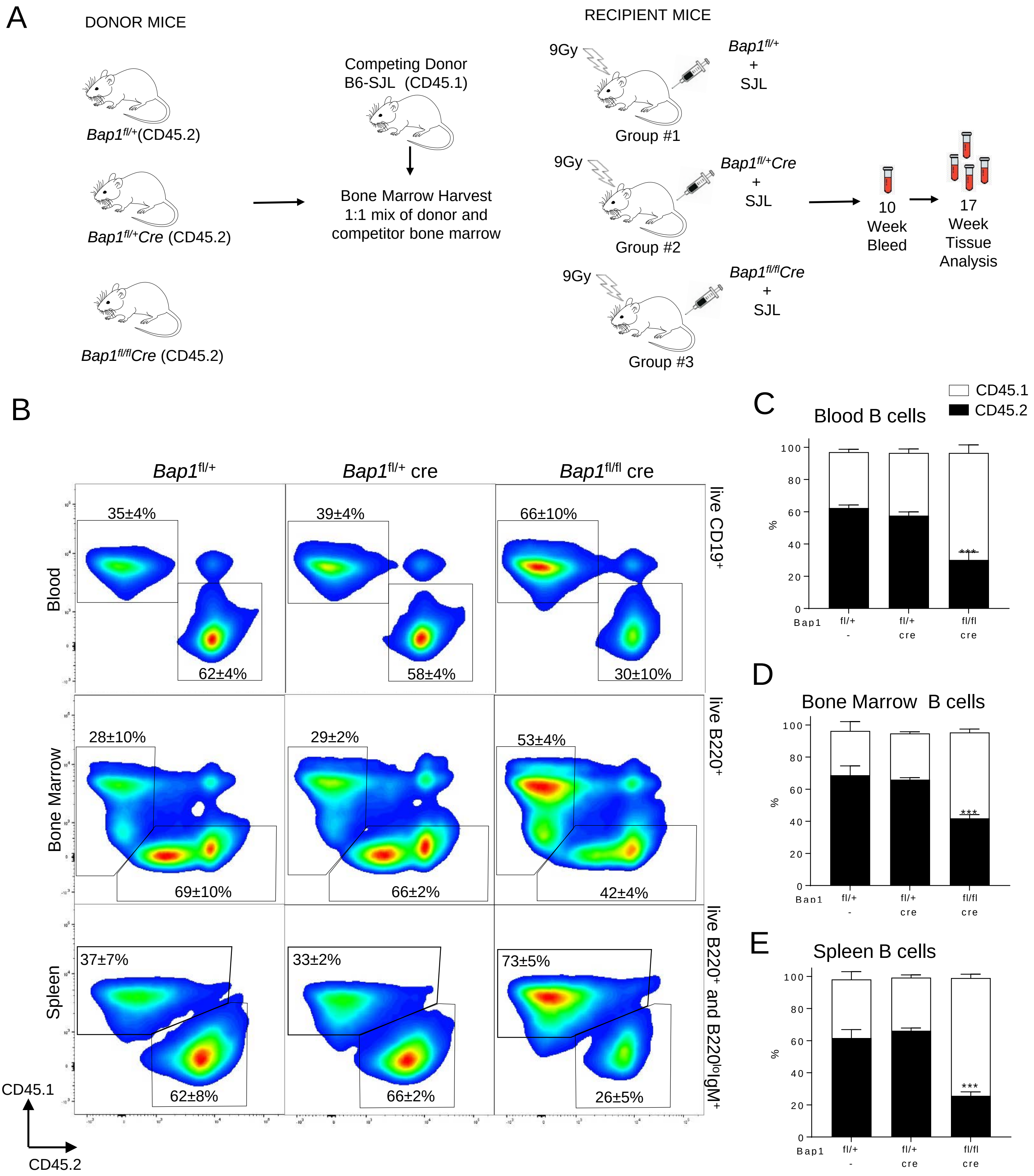

A
DONOR MICE
Bap1fl/+(CD45.2)
Bap1fl/+Cre (CD45.2)
Bap1fl/flCre (CD45.2)
Competing Donor
B6-SJL (CD45.1)
Bone Marrow Harvest
1:1 mix of donor and
competitor bone marrow
RECIPIENT MICE
9Gy
Bap1fl/+
+
SJL
Group #1
9Gy
Bap1fl/+Cre
+
SJL
Group #2
9Gy
Bap1fl/flCre
+
SJL
Group #3
10
Week
Bleed
17
Week
Tissue
Analysis
B
Bap1fl/+
Bap1fl/+ cre
Bap1fl/fl cre
Blood
35±4%
62±4%
39±4%
58±4%
66±10%
30±10%
live CD19+
Bone Marrow
28±10%
69±10%
29±2%
66±2%
53±4%
42±4%
live B220+
Spleen
37±7%
62±8%
33±2%
66±2%
73±5%
26±5%
live B220+ and B220loIgM+
CD45.1
CD45.2
C
Blood B cells
CD45.1
CD45.2
100
80
60
40
20
0
%
***
Bap1
fl/+
-
fl/+
cre
fl/fl
cre
D
Bone Marrow B cells
100
80
60
40
20
0
%
***
Bap1
fl/+
-
fl/+
cre
fl/fl
cre
E
Spleen B cells
100
80
60
40
20
0
%
***
Bap1
fl/+
-
fl/+
cre
fl/fl
cre

Supplemental Figure S4. Supporting data for flow cytometry analyses of *Bap1*$^{fl/fl}$ *Cre* mouse tissues. (A) Validation of the CD45.2^{+} and CD45.1^{+} B cell population gating. Bone marrow and spleen of control unmanipulated B6 (CD45.2^{+}) and B6-SJL (CD45.1^{+}) mice are analyzed for CD45.2 and CD45.1 marker expression, gating on live B220^{+} cells for the bone marrow, and on live B220^{+} or B220^{lo}IgM^{+} cells for the spleen. Mean cell frequency in the CD45.2^{+} and CD45.1^{+} gates is indicated. Related to Figure S3. **(B)** Validation of cell cycle analyses using Ki67 and histone H3S10p markers - isotype control staining for each marker is shown, gating on live large and live small pre-B cells (B220^{+}IgM^{-}IgD^{-}CD19^{+}CD43^{-}FSC$^{hi/lo}$). Mean cell frequency in the Ki67^{-} H3S10p^{-}, Ki67^{+}H3S10p^{-} and Ki67^{+}H3S10p^{+} gates is indicated. Related to Figure 3D. **(C)** Ba/F3 cell line stained for histone H3S10p, as a positive control validation for the staining; histograms are gated on live cells. Related to Figure 3D.

Figure S4

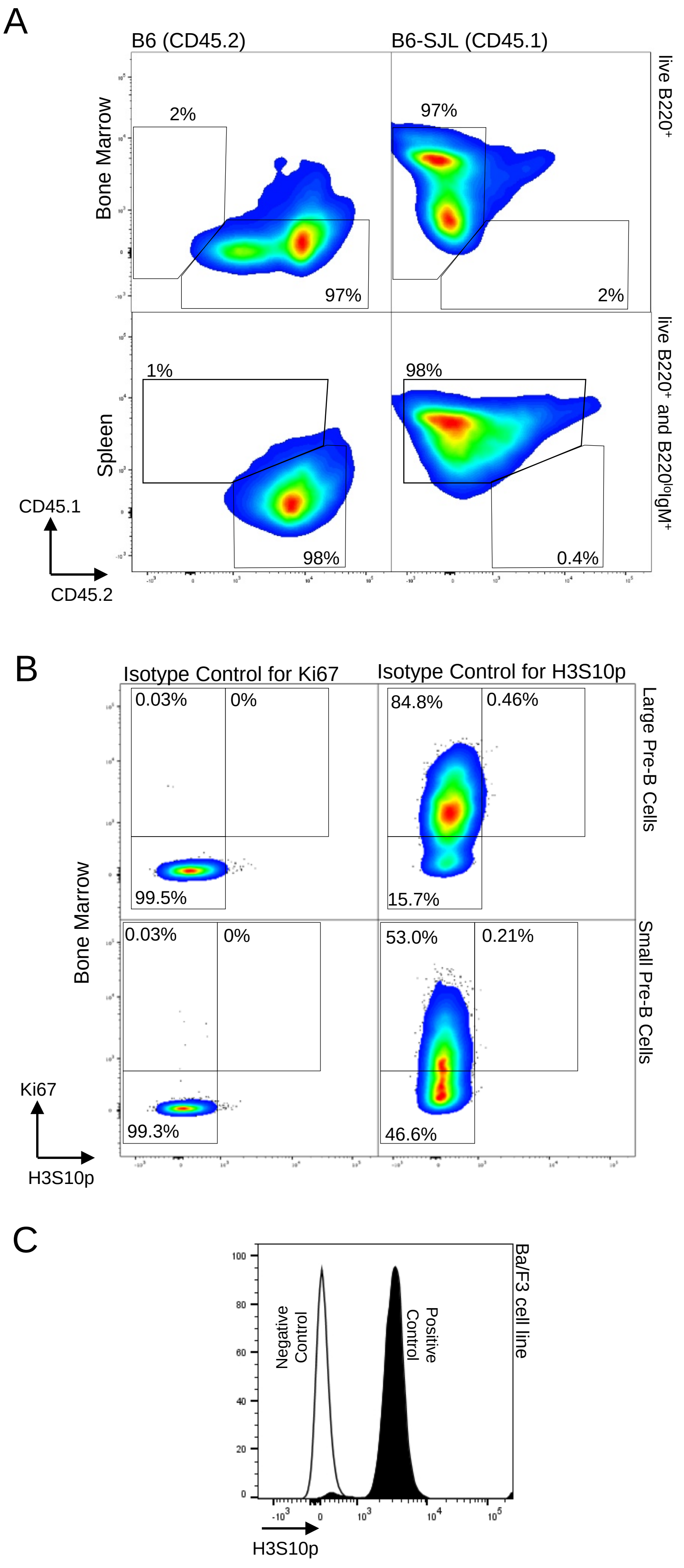

A
B6 (CD45.2)
B6-SJL (CD45.1)
live B220+
Bone Marrow
2%
97%
97%
2%
live B220+ and B220loIgM+
Spleen
1%
98%
98%
0.4%
CD45.1
CD45.2
B
Isotype Control for Ki67
Isotype Control for H3S10p
Large Pre-B Cells
Small Pre-B Cells
Bone Marrow
0.03%
0%
99.5%
84.8%
0.46%
15.7%
0.03%
0%
99.3%
53.0%
0.21%
46.6%
Ki67
H3S10p
C
Ba/F3 cell line
Negative Control
Positive Control
100
80
60
40
20
0
H3S10p

Supplemental Figure S5. Characterization of IL7Rα and pre-BCR expression on B cells in the bone marrow of *Bap1*$^{fl/fl}$ *Cre* mice. (A) Quantification of IL7Rα expression on B cell lineage cells in the bone marrow of *Bap1*$^{fl/fl}$ *Cre* and control mice. **(B)** Representative histograms showing IL7Rα staining of large and small pre-B cells of *Bap1*$^{fl/fl}$*Cre* and control mice. Cells are gated as live B220^{+}IgM^{-}IgD^{-}CD43^{-} FSC^{hi} or FSCl^{lo}, respectively. **(C)** Quantification of pre-B cell receptor (pre-BCR) expression on large and small pre-B cells of *Bap1*$^{fl/fl}$*Cre* and control mice. Cells are gated as live B220^{+}IgM^{-}IgD^{-}CD43^{-} FSC^{hi} or FSCl^{lo}, respectively. **(D)** Representative histograms showing pre-BCR staining or isotype control staining of large and small pre-B cells of *Bap1*$^{fl/fl}$*Cre* and control genotypes. n=3-4 mice were analyzed per genotype per experiment, and the data is consolidated from 2 experiments for IL7Rα and from one experiment for pre-BCR analyses; MFI - mean fluorescence intensity; statistical analyses were performed using ANOVA, *** $p<0.001$, or not significant if not indicated. Related to Figures 2-3.

Figure S5

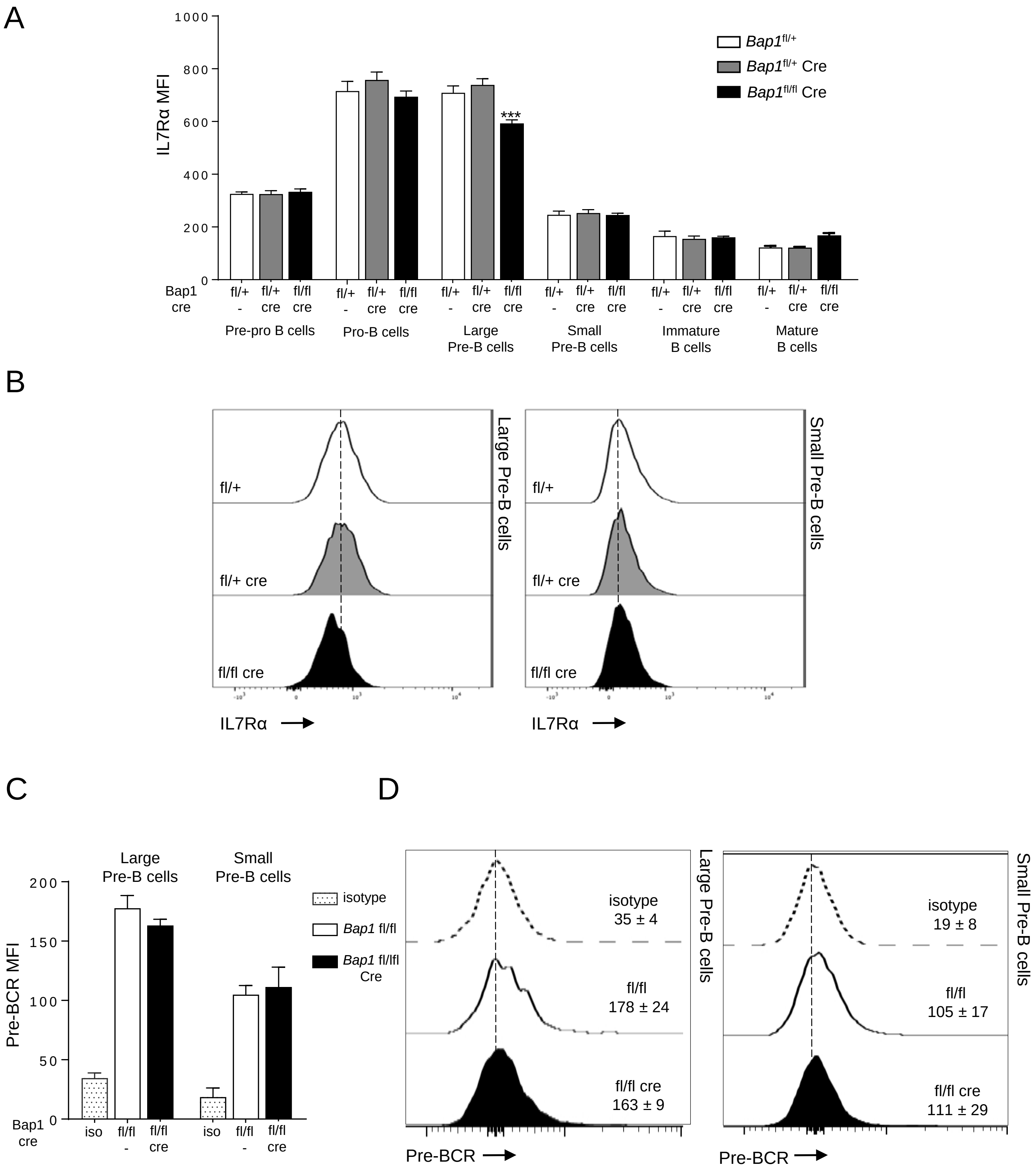
A
IL7Rα MFI
1000
800
600
400
200
0
Bap1fl/+
Bap1fl/+ Cre
Bap1fl/fl Cre
***
Bap1
cre
fl/+
-
fl/+
cre
fl/fl
cre
Pre-pro B cells
Pro-B cells
Large Pre-B cells
Small Pre-B cells
Immature B cells
Mature B cells
B
Large Pre-B cells
Small Pre-B cells
fl/+
fl/+ cre
fl/fl cre
IL7Rα
C
Large Pre-B cells
Small Pre-B cells
Pre-BCR MFI
200
150
100
50
0
isotype
Bap1 fl/fl
Bap1 fl/lfl Cre
Bap1
cre
iso
fl/fl
-
fl/fl
cre
D
isotype
35 ± 4
fl/fl
178 ± 24
fl/fl cre
163 ± 9
isotype
19 ± 8
fl/fl
105 ± 17
fl/fl cre
111 ± 29
Pre-BCR

Supplemental Figure S6. Characterization of γH2AX and p53 levels in B cells in the bone marrow of *Bap1*$^{\mathbf{fl/fl}}$ *Cre* mice. (A) Normal levels of γH2AX DNA damage marker and **(B)** p53 DNA damage response protein in *Bap1*$^{fl/fl}$*Cre* B cells. Cells were gated as B220^{+}IgM^{+}IgD^{+} for mature B cells, B220^{+}IgM^{+}IgD^{-} for immature B cells, B220^{+}IgM^{-}IgD^{-}CD43^{-} for pre-B cells, and B220^{+}IgM^{-}IgD^{-}CD43^{+} for pro-B cells and pre-pro-B cells. Bars represent mean ± SEM; n=4 mice were analyzed per genotype in total over two independent experiments; MFI – mean fluorescence intensity; statistical analyses were performed using ANOVA; not significant if significance is not indicated. **(C-D)** Representative histograms showing γH2AX and p53 staining of pre-B cells of *Bap1*$^{fl/fl}$*Cre* and control genotypes. Negative control histograms from the same cells stained with appropriate isotype control antibodies are also included. Related to Figures 2-3.

Figure S6

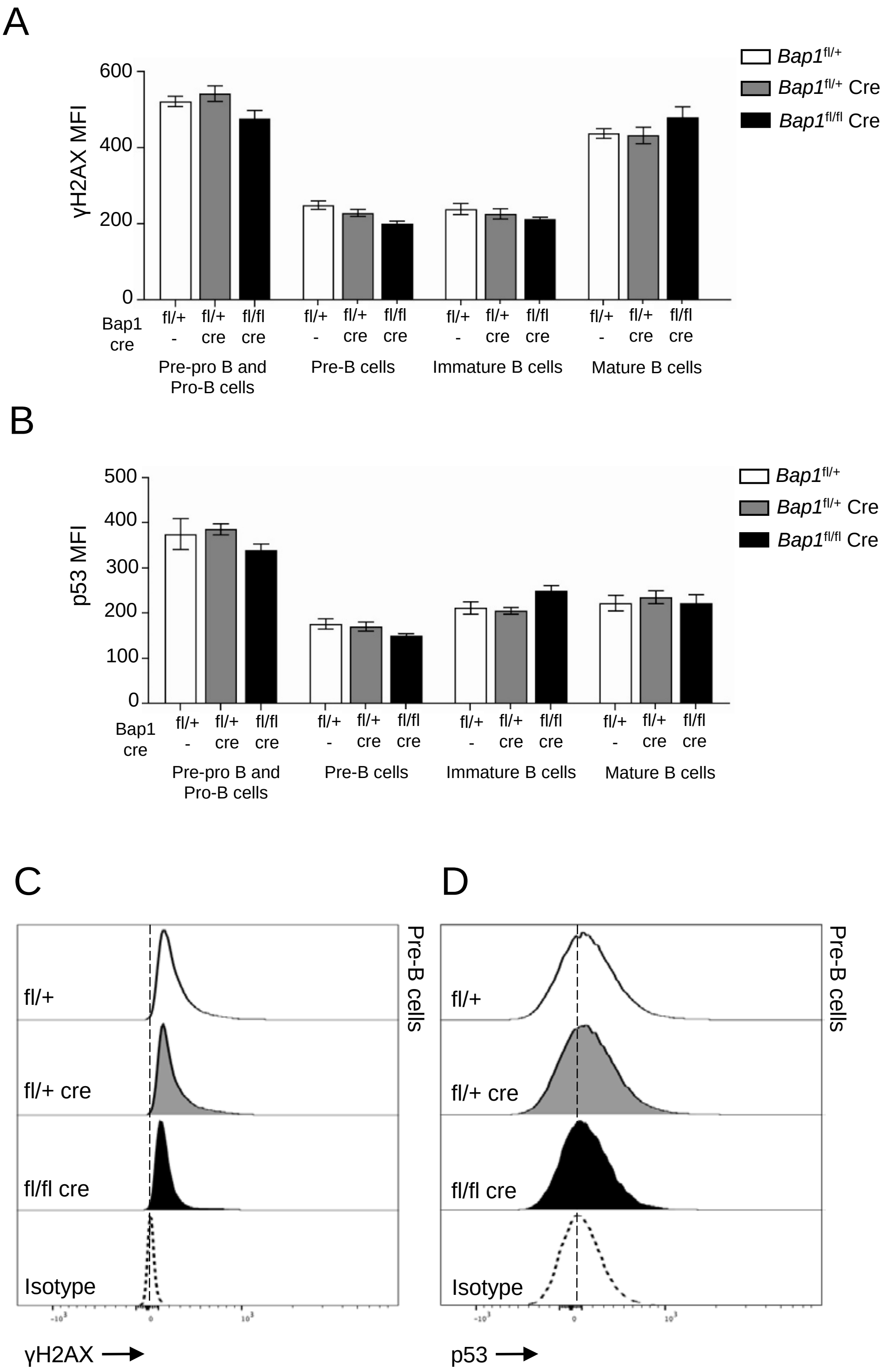
A
600
400
200
0
γH2AX MFI
Bap1
cre
fl/+ -
fl/+ cre
fl/fl cre
Pre-pro B and Pro-B cells
Pre-B cells
Immature B cells
Mature B cells
Bap1fl/+
Bap1fl/+ Cre
Bap1fl/fl Cre
B
500
400
300
200
100
0
p53 MFI
C
D
fl/+
fl/+ cre
fl/fl cre
Isotype
Pre-B cells
γH2AX
p53

Supplemental Figure S7. Further characterization of BAP1 RNA-Seq and ChIP-Seq data. **(A)** Dot plot showing the number of sequencing reads mapped to *Bap1* floxed exons 6-12 in RNA-seq data presented in Figure 4. Successful deletion of the *Bap1* floxed exons is observed in the $Bap1^{fl/fl}Cre$ cells. **(B)** Box plot showing histone H2AK119ub read intensities ±2.5kb to the gene proximal and gene distal BAP1 binding sites in the WT and two $Bap1^{\Delta/\Delta}$ (KO) B cell precursor cell lines. The *p*-values were calculated using Mann-Whitney U test. **(C-D)** Analyses of the co-localization of BAP1 binding sites from our ChIP-Seq datasets with the binding sites of BAP1 and other functionally related transcriptional regulators from previously published studies (Dey et al., 2012; Frangini et al., 2013; Micol et al., 2017; Kweon et al., 2019). **(C)** Heatmap showing the fold enrichment of genes linked to specific biological process GO-terms among the genes in vicinity of BAP1 binding sites in B cells (current study), ES cells (Kweon et al., 2019), and macrophages (Dey et al., 2012). GREAT analysis was performed on the pre-selected lists of "shared" and "cell-type specific" BAP1 binding sites (Zhang et al., 2008; McLean et al., 2010), with Basal plus extension option, searching for genes within 2kb upstream, 2kb downstream, and 200kb in distal. The top enriched biological processes were selected for further comparison. Gray boxes indicate non enriched terms. **(D)** Heat map showing the read intensities of various transcriptional regulators and histone marks around the 13,163 BAP1 binding sites identified in ChIP-Seq experiments from WT and triple-FLAG-tagged Ba/F3 B cell precursor cell lines. The sites are ranked based on their distances to the nearest gene transcriptional start site (TSS). The BAP1 ChIP-Seq from Ba/F3 cells represents our dataset and is described in Figure 6. The others are public datasets, downloaded and re-analyzed using our pipeline. These include the BAP1 ChIP-Seq from ES cells (Kweon et al., 2019), the BAP1, HCF1, and OGT ChIP-Seq from bone marrow derived macrophages (MΦ) (Dey et al., 2012), the ASXL1 ChIP-Seq from c-Kit^{+} hematopoietic progenitor cells (Micol et al., 2017), the RING1B, CBX7, EZH2, and USP16 ChIP-Seq from quiescent CD43^{-} resting splenic B cells (Frangini et al., 2013), and the YY1 ChIP-Seq from follicular B cells (CD19^{+}AA4^{-}CD21^{lo}CD23^{hi}) (Kleiman et al., 2016); all datasets are from mouse. **(E)** Genomic snapshots of selected genes that represent the putative direct transcriptional targets of BAP1 in pre-B cells. The genes are selected from Cluster V genes that are downregulated in expression in $Bap1^{fl/fl}Cre$ pre-B cells, carry proximal BAP1 binding site, and are functionally linked to cell proliferation and cell cycle progression. ChIP-Seq tracks for BAP1 and input DNA are shown on the top two lanes. The gene feature track is shown in the middle. Averaged RNA-Seq tracks for the pre-B cells are in the bottom three lanes, with fold changes comparing expression levels in $Bap1^{fl/fl}Cre$ and control $Bap1^{fl/+}$ samples indicated. The maximum data range of each track is indicated at the top-right corner of the track. Related to (A) Figure 4 and (B-E) Figures 6-7.

Figure S7

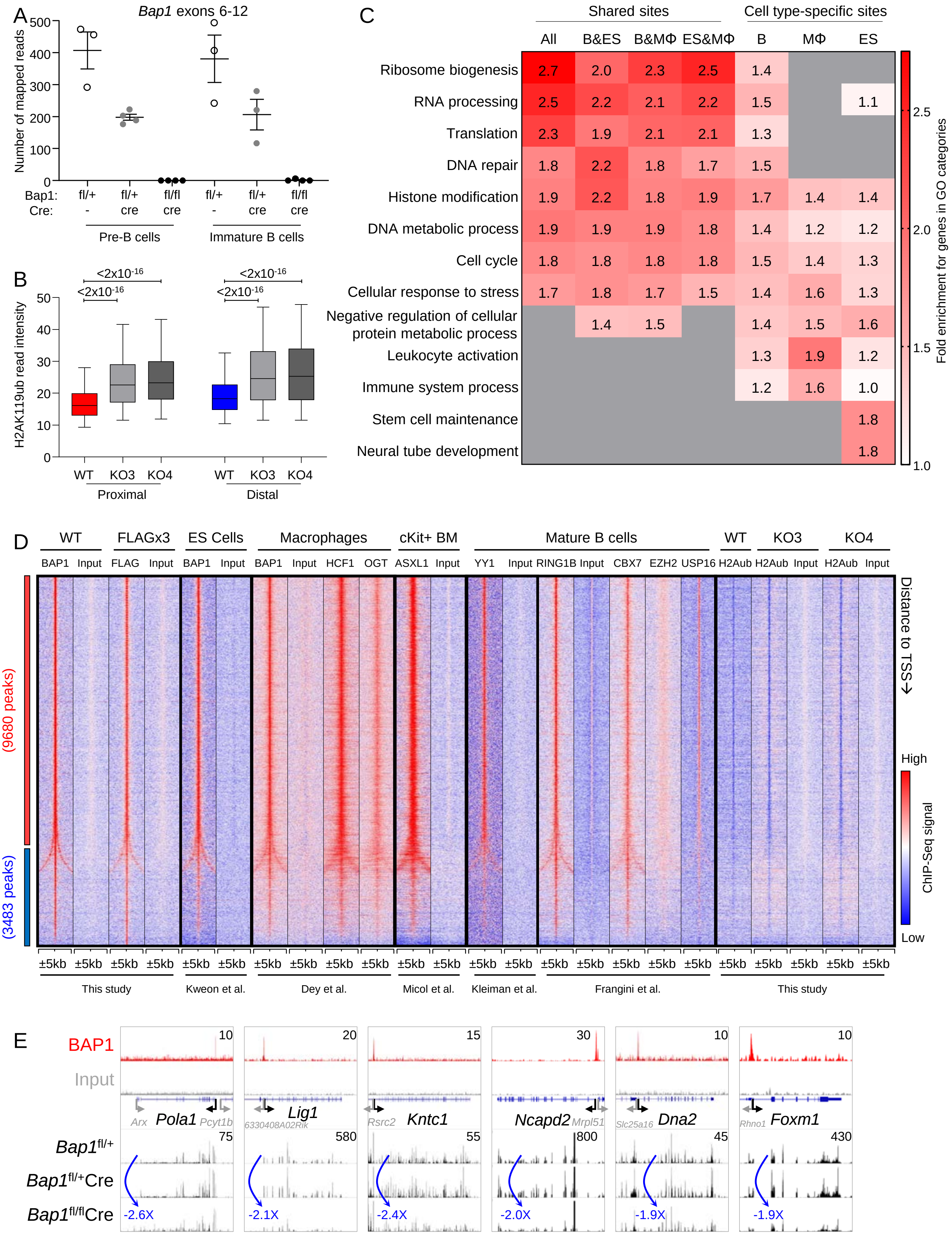
A
Bap1 exons 6-12
Number of mapped reads
500
400
300
200
100
0
Bap1: fl/+ fl/+ fl/fl fl/+ fl/+ fl/fl
Cre: - cre cre - cre cre
Pre-B cells
Immature B cells
B
<2x10-16
<2x10-16
<2x10-16
<2x10-16
H2AK119ub read intensity
50
40
30
20
10
0
WT KO3 KO4 WT KO3 KO4
Proximal
Distal
C
Shared sites
Cell type-specific sites
All B&ES B&MΦ ES&MΦ B MΦ ES
Ribosome biogenesis 2.7 2.0 2.3 2.5 1.4
RNA processing 2.5 2.2 2.1 2.2 1.5 1.1
Translation 2.3 1.9 2.1 2.1 1.3
DNA repair 1.8 2.2 1.8 1.7 1.5
Histone modification 1.9 2.2 1.8 1.9 1.7 1.4 1.4
DNA metabolic process 1.9 1.9 1.9 1.8 1.4 1.2 1.2
Cell cycle 1.8 1.8 1.8 1.8 1.5 1.4 1.3
Cellular response to stress 1.7 1.8 1.7 1.5 1.4 1.6 1.3
Negative regulation of cellular protein metabolic process 1.4 1.5 1.4 1.5 1.6
Leukocyte activation 1.3 1.9 1.2
Immune system process 1.2 1.6 1.0
Stem cell maintenance 1.8
Neural tube development 1.8
Fold enrichment for genes in GO categories
2.5
2.0
1.5
1.0
D
WT FLAGx3 ES Cells Macrophages cKit+ BM Mature B cells WT KO3 KO4
BAP1 Input FLAG Input BAP1 Input BAP1 Input HCF1 OGT ASXL1 Input YY1 Input RING1B Input CBX7 EZH2 USP16 H2Aub H2Aub Input H2Aub Input
Proximal (9680 peaks)
Distal (3483 peaks)
Distance to TSS
High
ChIP-Seq signal
Low
±5kb
This study
Kweon et al.
Dey et al.
Micol et al.
Kleiman et al.
Frangini et al.
This study
E
BAP1
Input
Bap1fl/+
Bap1fl/+Cre
Bap1fl/flCre
10 20 15 30 10 10
Arx Pola1 Pcyt1b
6330408A02Rik Lig1
Rsrc2 Kntc1
Ncapd2 Mrpl51
Slc25a16 Dna2
Rhno1 Foxm1
75 580 55 800 45 430
-2.6X -2.1X -2.4X -2.0X -1.9X -1.9X

Supplemental Table S1. Analyses of *Bap1*$^{fl/fl}$ *Cre* RNA-Seq and BAP1 ChIP-Seq datasets. Table attached as separate document in excel format.

(A-D) Analysis of the *Bap1*$^{fl/fl}$ *Cre* RNA-Seq dataset; related to Figures 4C-E. (**A)** List of genes differentially expressed in pre-B and immature B cells of *Bap1*$^{fl/fl}$ *Cre* relative to control *Bap1*$^{fl/+}$ mice; related to Figure 4D. Information provided for each gene includes: gene cluster, heatmap row number (Figure 4D), gene name, fold change, false discovery rate (FDR), and normalized counts per million. **(B)** Full list of genes expressed in pre-B and immature B cells of *Bap1*$^{fl/fl}$ *Cre*, *Bap1*$^{fl/+}$Cre, and control *Bap1*$^{fl/+}$ mice; the same information as in (A) is provided for each gene. **(C)** Normalized enrichment scores (NES) of 4,436 pre-established biological process signatures used in the gene set enrichment analysis (GSEA) and depicted in Figure 4C. In each column, positive values indicate biological process terms upregulated and negative values indicate terms downregulated in the comparison of *Bap1*$^{fl/fl}$ *Cre* to control pre-B cells. **(D)** Gene ontology enrichment analyses showing the enriched biological process (BP) terms for each cluster of differentially expressed genes from Figures 4D-E.

(E-F) Analysis of the BAP1 ChIP-Seq dataset; related to Figures 6A-B. **(E)** List of BAP1 binding peaks identified in the B cell precursor cell line Ba/F3. Information provided for each binding peak includes: peak group, peak location, distance to the nearest gene transcription start site (TSS), normalized read intensities ±100bp around peak summit, whether this binding peak was also identified in previous BAP1 ChIP-Seq studies performed in ES cells and macrophages, and whether it overlaps with the binding sites of other functionally related transcriptional regulators (Dey et al., 2012; Frangini et al., 2013; Micol et al., 2017; Kweon et al., 2019). **(F)** Gene Ontology analysis of the nearest genes to each BAP1 binding peak in the gene proximal and gene distal groups, performed on the GREAT website (*http://great.stanford.edu/*) (McLean et al., 2010), with basal plus extension option, searching for genes within 2kb upstream, 2kb downstream, and 200kb in distal; related to Figure 6B.

(G-H) Consolidation of *Bap1*$^{fl/fl}$ *Cre* RNA-Seq and BAP1 ChIP-Seq data; related to Figure 7A-B. Putative BAP1-regulated genes are identified as having BAP1-binding peaks in ChIP-Seq data, and significant dysregulation in expression in *Bap1*$^{fl/fl}$ *Cre* relative to control *Bap1*$^{fl/+}$ cells in the RNA-Seq data. **(G)** List of putative BAP1-regulated genes, with information provided including: dysregulated gene cluster, BAP1 peak set, search window, whether the gene is linked to gene ontology (GO) terms related to “cell cycle”, “DNA replication”, and “cell division”, the gene dysregulation status, whether the binding peaks of other functionally related transcriptional regulators were also identified near the BAP1 peak in previously published studies (Dey et al., 2012; Frangini et al., 2013; Micol et al., 2017; Kweon et al., 2019), and detailed gene expression information. BAP1 binding peaks within the search window for each dysregulated gene are shown. Note that a gene can have more than one peak within the search window. **(H)** Gene ontology enrichment analyses showing the enriched biological process (BP) terms for each cluster of putative BAP-regulated genes; related to Figure 7B.

Supplemental References

Allman, D., and Pillai, S. (2008). Peripheral B cell subsets. *Curr Opin Immunol* 20(2)**,** 149-157. doi: 10.1016/j.coi.2008.03.014.

Baumgarth, N. (2011). The double life of a B-1 cell: self-reactivity selects for protective effector functions. *Nat Rev Immunol* 11(1)**,** 34-46. doi: 10.1038/nri2901.

Dey, A., Seshasayee, D., Noubade, R., French, D.M., Liu, J., Chaurushiya, M.S., et al. (2012). Loss of the tumor suppressor BAP1 causes myeloid transformation. *Science* 337(6101)**,** 1541-1546. doi: 10.1126/science.1221711.

Frangini, A., Sjoberg, M., Roman-Trufero, M., Dharmalingam, G., Haberle, V., Bartke, T., et al. (2013). The aurora B kinase and the polycomb protein ring1B combine to regulate active promoters in quiescent lymphocytes. *Mol Cell* 51(5)**,** 647-661. doi: 10.1016/j.molcel.2013.08.022.

Kleiman, E., Jia, H., Loguercio, S., Su, A.I., and Feeney, A.J. (2016). YY1 plays an essential role at all stages of B-cell differentiation. *Proc Natl Acad Sci U S A* 113(27)**,** E3911-3920. doi: 10.1073/pnas.1606297113.

Kweon, S.M., Chen, Y., Moon, E., Kvederaviciute, K., Klimasauskas, S., and Feldman, D.E. (2019). An Adversarial DNA N(6)-Methyladenine-Sensor Network Preserves Polycomb Silencing. *Mol Cell* 74(6)**,** 1138-1147 e1136. doi: 10.1016/j.molcel.2019.03.018.

McLean, C.Y., Bristor, D., Hiller, M., Clarke, S.L., Schaar, B.T., Lowe, C.B., et al. (2010). GREAT improves functional interpretation of cis-regulatory regions. *Nat Biotechnol* 28(5)**,** 495-501. doi: 10.1038/nbt.1630.

Micol, J.B., Pastore, A., Inoue, D., Duployez, N., Kim, E., Lee, S.C., et al. (2017). ASXL2 is essential for haematopoiesis and acts as a haploinsufficient tumour suppressor in leukemia. *Nat Commun* 8**,** 15429. doi: 10.1038/ncomms15429.

Wells, S.M., Kantor, A.B., and Stall, A.M. (1994). CD43 (S7) expression identifies peripheral B cell subsets. *J Immunol* 153(12)**,** 5503-5515.

Zhang, Y., Liu, T., Meyer, C.A., Eeckhoute, J., Johnson, D.S., Bernstein, B.E., et al. (2008). Model-based analysis of ChIP-Seq (MACS). *Genome biology* 9(9)**,** R137. doi: 10.1186/gb-2008-9-9-r137.
